# Dissecting the ROS signalling component of salinity tolerance: tissue-specific K^+^/Na^+^ homeostasis in quinoa and spinach roots

**DOI:** 10.1093/jxb/erag021

**Published:** 2026-01-19

**Authors:** Mohsin Tanveer, Muhammad Saqib Bilal, Zhong-Hua Chen, Lei Wang, Sergey Shabala

**Affiliations:** Key Laboratory of Ecological Safety and Sustainable Development in Arid Lands, Xinjiang Institute of Ecology and Geography, Chinese Academy of Sciences, Urumqi, Xinjiang, China; Key Laboratory of Ecological Safety and Sustainable Development in Arid Lands, Xinjiang Institute of Ecology and Geography, Chinese Academy of Sciences, Urumqi, Xinjiang, China; School of Agriculture, Food and Wine, University of Adelaide, Glen Osmond, SA 5064, Australia; Key Laboratory of Ecological Safety and Sustainable Development in Arid Lands, Xinjiang Institute of Ecology and Geography, Chinese Academy of Sciences, Urumqi, Xinjiang, China; School of Biological Sciences and ARC Training Centre for Smart and Sustainable Horticulture, University of Western Australia, Perth, Australia; International Research Centre for Environmental Membrane Biology, Foshan University, Foshan 528000, China; Universidade Federal do Ceará, Brazil

**Keywords:** Glycophyte, halophyte, hormonal crosstalk, K^+^/Na^+^, spatiotemporal regulation

## Abstract

This study combines electrophysiological, imaging, and molecular techniques to compare reactive oxygen species (ROS)-mediated K^+^/Na^+^ regulation in the root elongation zone (EZ) and mature zone (MZ) of halophytic quinoa (*Chenopodium quinoa*) and glycophytic spinach (*Spinacia oleracea*). Under salinity stress, quinoa exhibited transient ROS (H_2_O_2_) accumulation followed by rapid recovery, whereas spinach showed prolonged oxidative stress and severe ionic imbalance in roots. Quinoa plants avoided cytosolic Na^+^ toxicity by excluding Na^+^ via the up-regulation of *salt overly sensitive* (*SOS1*) genes and enhanced vacuolar sequestration via *NHX*. Quinoa maintained K^+^ homeostasis under ROS through biphasic regulation linked to tissue-specific expression of K^+^ transporter genes *GORK*, *AKT1*, *HAK5*, and *KEA*, while spinach experienced a sustained K^+^ loss. Transcriptomic analysis revealed robust induction of *MAPK* signalling and ethylene-related genes in quinoa, contrasting with the reliance of spinach on abscisic acid and delayed antioxidant responses. Overall, the differential sensitivity of root zones was attributed to the spatially restricted ROS signalling in quinoa, which fine-tunes ion transporter activity, while spinach showed excessive ROS production and K^+^ loss. These results demonstrate that the oxidative tolerance of quinoa arises from coordinated ROS–hormone–transporter interactions in a highly tissue-specific manner, providing a mechanistic framework for improving crop resilience.

## Teaser

Superior salinity stress tolerance in quinoa originates from tissue-specific ROS–hormone–transporter interactions that regulate expression of K^+^ transporter genes *GORK*, *AKT1*, *HAK5*, and *KEA*, thus mediating K^+^ homeostasis.

## Introduction

Soil salinity represents one of the most prevailing abiotic stresses limiting global agricultural productivity (Razzaq *et al*., 2021) while affecting ∼20% of irrigated land worldwide (FAO, 2024). At the physiological level, salinity stress disrupts cellular ion homeostasis by increasing the accumulation of toxic sodium ions (Na^+^) and reducing potassium (K^+^) content, leading to widespread metabolic dysfunctions (Shabala and Cuin, 2008; Khan *et al*., 2020). While all plants experience these ionic imbalances, halophytic species, including quinoa (*Chenopodium quinoa*), demonstrate remarkable resilience through sophisticated mechanisms that have evolved to maintain optimal cytosolic K^+^/Na^+^ ratios under saline conditions (Shabala, 2013; Bose *et al*., 2017; Rasouli *et al*., 2023; Tanveer *et al*., 2024a; Rawat *et al*., 2025).

A key secondary effect of salinity-induced ionic imbalance is oxidative stress, driven by excessive production of reactive oxygen species (ROS) associated with elevated Na^+^ levels in the cytosol (Isayenkov and Maathuis, 2019). Having said that, ROS (especially H_2_O_2_) exhibit dual roles as either cytotoxic agents or signalling molecules depending on their concentration (Tanveer and Ahmed, 2020; Mittler *et al*., 2022). While ROS production is a universal signalling mechanism activated by diverse abiotic and biotic stresses, the specific role of ROS in regulating ionic homeostasis, particularly Na^+^ dynamics and the critical K^+^/Na^+^ balance, remains a pivotal yet underexplored aspect of salinity tolerance. The resolution of this paradox potentially lies in the precise spatiotemporal regulation of ROS production and scavenging, thereby shaping ROS signalling. Notably, halophytes appear to have evolutionarily refined their ROS signalling networks for greater control, often exhibiting lower and more transient ROS bursts compared with glycophytes (Ellouzi *et al*., 2011; Bose *et al*., 2014; Srivastava *et al*., 2015), suggesting an evolutionary refinement of ROS signalling networks in salt-tolerant species (Singh *et al*., 2024). A critical downstream effect of ROS is the direct perturbation of cytosolic K^+^/Na^+^ homeostasis, where excessive ROS can activate K^+^ efflux channels (e.g. GORK) and Na^+^-permeable non-selective cation channels (NSCCs), exacerbating ionic stress (Demidchik, 2014). Salt-tolerant species reduce this K^+^ efflux via enhanced ROS scavenging capacity and/or reduced ion channel sensitivity to ROS (Wu *et al*., 2015a; H. Wang *et al*., 2025), without compromising ROS signalling. The specific details of this process remain vague.

Crucially, ROS signalling and ion homeostasis must be viewed in a tissue-specific context. Plant roots are functionally compartmentalized, with different zones probably having distinct ionic priorities. The elongation zone (EZ), characterized by rapidly expanding cells, may prioritize K^+^ retention and Na^+^ exclusion to maintain turgor pressure and membrane potential required for growth, while the root mature zone (MZ), with its developed vacuoles and endodermal barriers, may specialize in Na^+^ sequestration (Verbelen *et al*., 2006; Baluška *et al*., 2010; Gao *et al*., 2015; Gill *et al*., 2018). Such spatial regulation of different ion transporters and channels may be causally linked to the spatial regulation of ROS production. For instance, halophytes, unlike glycophytes, exhibit highly coordinated tissue- and cell-specific responses to oxidative and ionic stress (Yang *et al*., 2010; Zhou *et al*., 2020; Tang *et al*., 2021), while ROS production is not uniform but occurs in distinct cellular compartments and tissues, often serving as localized signals rather than indiscriminate toxins (Sachdev *et al*., 2021; Kesawat *et al*., 2023). Critically, the root apex exhibits rapid H_2_O_2_ accumulation upon salt exposure, which can trigger adaptive responses such as cell wall remodelling (Miller *et al*., 2010; Mittler *et al*., 2011) and targeted ion transport (S. Shabala *et al*., 2016). Conversely, excessive ROS production in the MZ (representing the bulk of the root) or leaf tissues often correlates with oxidative damage (Bernstein *et al*., 2010; Zhang *et al*., 2017), emphasizing the importance of spatial regulation.

Temporal dynamics of ion homeostasis mechanisms add another layer of complexity to salinity stress responses, as transcript levels of ion channels/transporters regulating K^+^/Na^+^ homeostasis exhibit distinct expression patterns over time following salt exposure, with some responses occurring within hours while others develop over days, regardless of their tolerance levels (Zhou *et al*., 2020; Wang *et al*., 2022). ROS signalling further intertwines with hormonal pathways to fine-tune these responses. For instance, ethylene (ETH) has emerged as particularly important for salinity tolerance, promoting ROS scavenging through activating antioxidant systems while simultaneously enhancing the activity of *SOS1* and *NHX* transporters (Jiang *et al*., 2013; Lang *et al*., 2020). Conversely, abscisic acid (ABA) reduces Na^+^ accumulation in shoots by limiting transpiration-driven water flow (Cabot *et al*., 2009; Gurmani *et al*., 2013) while simultaneously triggering ROS production via NADPH oxidases [respiratory burst oxidase homologues (RBOHs)] to modulate root growth (Zhang *et al*., 2009; Xie *et al*., 2016; Majeed *et al*., 2023). Genetic evidence highlights the antagonism between ABA and ETH: the ABA-hypersensitive mutant *abo6* in Arabidopsis exhibits suppressed ETH signalling and elevated ROS accumulation in root tips (Yang *et al*., 2014; Yu *et al*., 2019), underscoring their crosstalk in growth regulation. Although ABA-induced ROS disrupts redox homeostasis, plants counterbalance this through ROS-scavenging systems (S. Li *et al*., 2022). However, since ROS signals are transiently required to activate stress cascades, the spatiotemporal coordination of this hormone–ROS crosstalk across different root tissues remains poorly understood.

Several unresolved questions persist. (i) How do ROS production and signalling dynamics differ between root zones of halophytes and glycophytes? (ii) How is subcellular ion partitioning coordinated by localized ROS signals? (iii) What is the role of hormone–ROS interactions in modulating ion transport in a tissue-specific manner? (iv) What are the overarching transcriptional networks that integrate these responses?

**Fig. 1.**
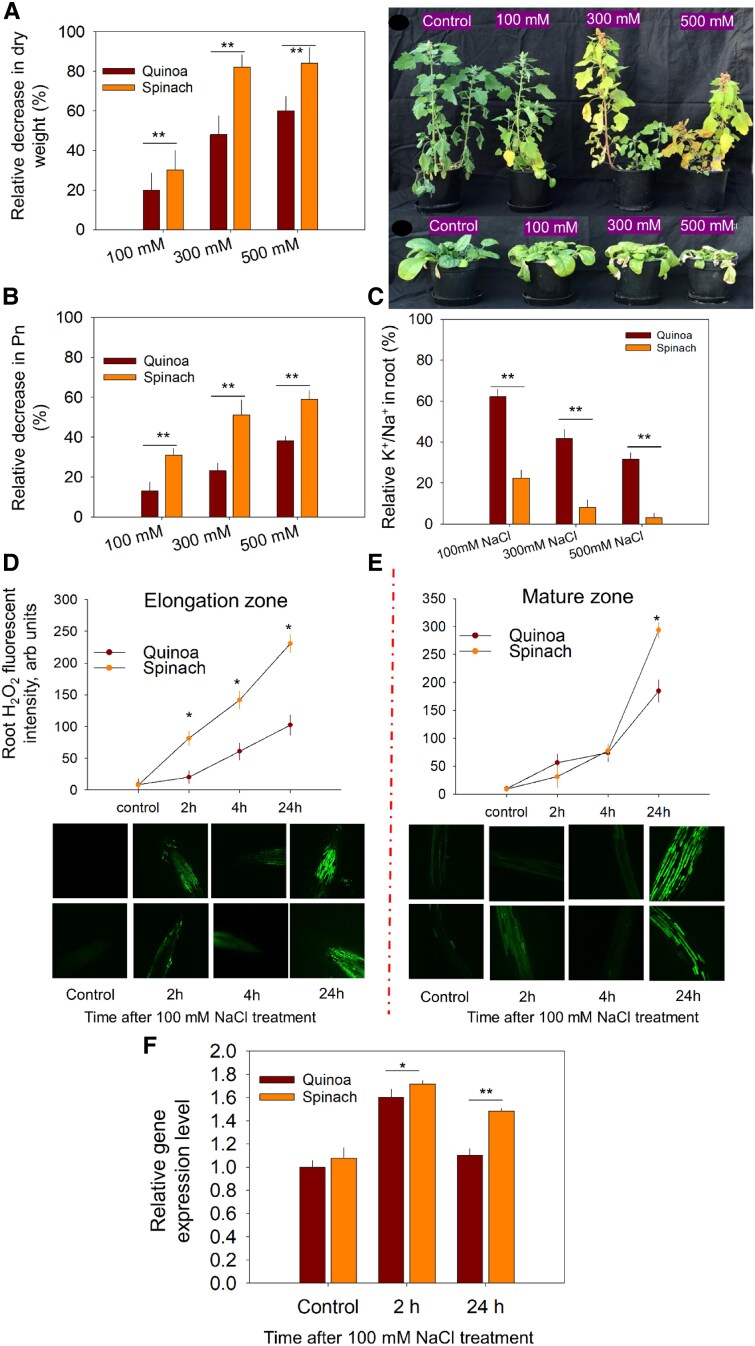
Response of quinoa and spinach under salinity treatment. (A) Relative decrease in plant DW under salinity (% of control), (B) relative decrease in photosynthesis (Pn) (% of control), (C) relative K^+^/Na^+^ in roots under different salinity levels, (D and E) quantification of root ROS fluorescent intensity in the root elongation and mature zone in response to 100 mM NaCl treatment, (F) expression of *RBOHA* in quinoa and spinach roots at 2 h and 24 h after NaCl stress. Data are the mean ±SE (*n*=3) for (A), (B), (C), and (F), while for (D) and (E), the data are the mean ±SE [*n*=160–240; 15–30 cells analysed for at least 7–9 individual roots (biological replicates)]. The error bars are SEs. Asterisks show the significant difference between species at the individual time point of stress at *P≤0.05 and **P≤0.01, respectively.

To address these questions, we employed a targeted approach using two contrasting species within the same family. We selected quinoa (*Chenopodium quinoa*), a facultative halophyte, and spinach (*Spinacia oleracea*), a glycophyte from the same *Amaranthaceae* family. This choice allows for a more nuanced comparison, as any profound differences in ROS signalling mechanisms are likely to represent core adaptive traits of halophytism, rather than general family-wide characteristics. We first established the physiological context of salinity tolerance in quinoa and spinach (see Fig. 1). We then used exogenous H_2_O_2_ application as a precise tool to dissect the core ROS signalling module, independent of the complex ionic and osmotic effects of long-term NaCl stress. By integrating electrophysiological, fluorescence imaging, and molecular techniques, this strategy allowed us to directly compare the spatiotemporal regulation of ROS signalling and K^+^/Na^+^ homeostasis in the EZ and MZ of the root of quinoa and spinach. Our results demonstrate that ROS may act as a pivotal signal that differentially regulates K^+^/Na^+^ homeostasis across root zones (EZ versus MZ), driving cell-specific redistribution of ions between the cytosol and the vacuole. This spatial regulation is mediated by ROS-induced activation of mitogen-activated protein kinase (MAPK) and RBOH pathways, phytohormonal crosstalk (ETH versus ABA), and antioxidant activation, culminating in transcriptional reprogramming of ion transporter genes (e.g. *SOS1*, *HKT1*, *HAK5*, and *NHX*) to optimize their operation.

## Materials and methods

### Plant material and growth conditions

Seeds of *C. quinoa* L. cv 5206 (quinoa) were donated by Professor Sven-Erik Jacobsen (University of Copenhagen, Denmark) and later propagated using facilities of the Tasmanian Institute of Agriculture (Hobart, Tasmania). Seeds of *S. oleracea* L. cv. Greenfeast (spinach) were purchased from a local supplier (Hollander Imports, Hobart, Australia). Seeds were surface sterilized using 5% commercial bleach for 5–10 min and then thoroughly rinsed with double-distilled water five times before planting. In glasshouse experiments, quinoa and spinach were grown in 4 litre pots with the standard potting mixture (Tanveer *et al*., 2024a) under normal growth conditions (day/night temperature 26/18 °C; day length 14 h; relative humidity 65%). When plants were 3 weeks old, salinity treatments (100, 300, and 500 mM NaCl; added to irrigation water) were applied for 10 d, followed by agronomic (relative DW) and physiological (photosynthesis and K^+^ and Na^+^ contents) assessments. The experiment was conducted in a randomized complete block design, with six biological replications per treatment, six pots per biological replication, and three plants per pot. In laboratory experiments, seeds were germinated in a Petri dish for 2 d before being transferred to a hydroponic setup with half-strength Hoagland’s nutrient solution. All experiments were conducted using 7-day-old seedlings, which were cultivated in controlled growth chambers under the following conditions: a 16 h light/8 h dark photoperiod, day/night temperatures of 25/20 °C, and 75% relative humidity.

### Measurements

Harvested samples were oven-dried at 65 °C for 48 h, and DW was measured using a digital analytical balance (Mettler Toledo, TLE3002). Prior to harvesting, the net CO_2_ assimilation rate (Pn) was measured in intact leaves using an infrared gas analyser (IRGA) system (Bioscientific, Hoddesdon, UK). Measurements were taken between 12.00 h and 14.00 h on clear days under ambient photon flux density. K^+^ and Na^+^ contents in roots were determined from digested samples using a flame photometer (PFP7, Jenway; Bibby Scientific Ltd, Stone, UK) and inductively coupled plasma emission spectrometry (Prodigy XP, LEEMAN, Inc., USA), respectively.

For ETH quantification, fresh root samples (2–3 roots, ∼1 g FW) were carefully detached to minimize mechanical damage and placed in 100 ml gastight glass vessels, sealed with specialized lids designed for gas sampling, and incubated at room temperature for 48 h. A 1 ml gas sample was collected from the headspace using a sterile syringe (Braun Melsungen AG) and analysed via flame ionization GC (Varian 3700, Palo Alto, CA, USA). ETH concentrations were determined using a standard calibration curve and expressed as μmol g^−1^ FW h^−1^ (Li *et al*., 2014). ABA quantification was performed using an ABA Immunoassay Detection Kit (Sigma-Aldrich) following the manufacturer’s protocol. Absorbance was measured at 450 nm using a microplate reader, and ABA concentrations were calculated based on a standard curve, normalized to FW.

For proline measurement, 0.5 g of fresh roots were homogenized in 5% sulfosalicylic acid, and the supernatant was extracted after centrifugation for 6 min at 6000 *g*. Extracted supernatant was then mixed with 2 ml of gallic acid and 2 ml of ninhydrin, and heated at 70 °C for 1 h using a hot water bath. After heating, the supernatant was left to cool for another 1 h, and then the absorbance of proline was estimated at 520 nm spectrophotometrically (Tanveer *et al*., 2024a). The total phenol content was determined spectrophotometrically at 760 nm, using the Folin–Ciocalteu reagent. A 125 μl aliquot of extract was combined with 2.5 ml of Folin–Ciocalteu reagent, 375 μl of distilled water, and 2 ml of sodium carbonate. The mixture was incubated in a water bath at 50 °C for 5 min and, after cooling at room temperature, the optical density was measured with a spectrophotometer (Camspec *M106* spectrophotometer, Leeds, UK). Gallic acid was used to develop standard curves (Petridis *et al*., 2012; Jia *et al*., 2024). The catalase (CAT) and ascorbate peroxidase (APX) activities were measured in both root zones at 2 h and 24 h post-ROS stress, according to the manufacturer’s instructions for the appropriate kit (Beyotime, Shanghai, China).

### Laser scanning confocal microscopy for ion fluorescence detection

Fluorescent H_2_O_2_, K^+^, and Na^+^ dyes were used to visualize their distribution in various root zones. Experiments were conducted on 7-day-old roots measuring signals from the EZ and MZ. H_2_O_2_ was detected at 2 h and 24 h post-100 mM NaCl using 20 µM 2′,7′-dichlorofluorescein diacetate in a measuring buffer (10 mM KCl, 5 mM Na^+^, 5 mM Ca^2+^-MES, pH 6.1) with 30 min incubation. At the same time, Na^+^ and K^+^ fluorescence intensity was measured at 2 h and 24 h post-5 mM H_2_O_2_ using CoroNa Green AM dye (Invitrogen, Eugene, OR, USA) and Asante Potassium Green-2 (TEFLabs, Austin, TX, USA), respectively. Stock solutions (1 mM) of each dye were prepared in DMSO and each diluted to 20 µM in buffer solutions (without Na^+^), followed by 3 h dark incubation at 23 °C. After washing, stained roots were imaged using a Leica microscope with ×10 (NA 0.35) and ×40 oil immersion (NA 0.75) objectives for whole-root and intracellular analysis, respectively. Both dyes showed excitation/emission maxima near 490/520 nm. Images were acquired using Leica Application Suite X and analysed with LAS AF Lite software. For intracellular measurements, line scans across regions of interest were performed, with background signals subtracted from cytosolic and vacuolar readings. Fluorescence intensity profiles (in arbitrary units) were generated and plotted in SigmaPlot v10. Mean values for each compartment were calculated by correlating fluorescence patterns with root morphology from light microscopy images. Total cellular fluorescence was quantified from 20–35 cells per root across 10 replicates, while intracellular analysis examined 70–300 cells per genotype, with background-corrected values averaged for each treatment. A representative image from 10 replicates is presented in the Results, following established protocols (Bonales-Alatorre *et al*., 2013; Wu *et al*., 2015a; Wu *et al*., 2020).

For measuring K^+^ and Na^+^ fluorescence intensity in ETH- and ABA-pre-treated roots, 7-day-old quinoa and spinach roots were incubated in 50 µM ethephon (CAS no. 16672870, Sigma Aldrich) and 0.5 µM ABA (CAS no. 21293298, Sigma Alrich) for 6 h and transferred to 1/2 Hoagland solution with 5 mM H_2_O_2_ (CAS no. 7722841, Sigma Aldrich). Following 2 h and 24 h ROS treatments, roots were gently washed in double-distilled water and then the K^+^ and Na^+^ fluorescence intensity in different root zones was measured.

### Non-invasive ion flux measurement experiments

Net K^+^ and Na^+^ fluxes were measured from apical (2 mm from the tip) and mature (∼15 mm) root zones using the non-invasive microelectrode ion flux estimation (MIFE) technique (University of Tasmania), as described elsewhere (L. Shabala *et al*., 2016; Zhu *et al*., 2017). K^+^-selective electrodes were made using a standard K^+^ ionophore cocktail (Sigma-Aldrich 60031), while Na^+^ measurements employed specialized calixarene-based microelectrodes (Jayakannan *et al*., 2011). For measurements, seedlings were immobilized in chambers and pre-conditioned in basal slat mediun (BSM; 30 min for K^+^; overnight in 20 mM NaCl-containing BSM for Na^+^). After incubation, roots were washed with deionized water. Electrodes were positioned 40 µm from root surfaces using a 3D micromanipulator and moved between 40 µm and 120 µm in 5 s half-cycles. Following 5 mM H_2_O_2_ treatment, transient Na^+^ fluxes were recorded for 15 min, while the mean value of Na^+^ flux was calculated to measure Na^+^ flux (30 min) after applying 5 mM H_2_O_2_. The K^+^ fluxes were monitored continuously for 30 min.

### Transcriptomics

Plants were subjected to 5 mM H_2_O_2_ stress for 2 h. Apical (0–5 mm from the tip) and mature (5–20 mm) root segments were harvested. Total RNA was isolated using a TRIzol kit (Invitrogen, Carlsbad, CA, USA) with three biological replicates for each treatment. Purity, concentration, and integrity of RNA sample were examined by NanoDrop, Qubit 2.0, and Agilent 2100 (Agilent Technologies, CA, USA), respectively. Library construction and RNA sequencing (RNA-seq) were conducted by the BMKgene Bioinformatics Institute (Beijing, China) using the high-throughput sequencing platform with PE150 mode. Raw reads were filtered to remove low-quality reads and cleaned by removing adapters. The quality control of raw sequencing was conducted using Trim Galore (http://www.bioinformatics.babraham.ac.uk/projects/trim_galore/). Low-quality bases (quality value <20) were omitted, and if the quality value of the residual sequence was still <10, the entire sequence was removed; otherwise, the read was retained. The cleaned high-quality reads were then mapped to the quinoa and spinach reference genomes using HISAT2. StringTie was applied to assemble the mapped reads. Fragments per kilobase of transcript sequence per million base pairs sequenced (FPKM) were calculated using the StringTie flow algorithm.

Differential expression analysis of RNA-seq between control and ROS for quinoa and spinach was performed using the DESeq2 package. The resulting *P*-values were adjusted using the Benjamini and Hochberg’s approach for controlling the false discovery rate (FDR). Genes with FDR <0.05 and (log_2_fold change) >1 were assigned as differentially expressed genes (DEGs), and gene expression levels were reported in FPKM values. The DEGs were analysed using Kyoto Encyclopedia of Genes and Genomes (KEGG) pathway enrichment to identify biologically relevant pathways. The enrichment factor was calculated to assess the degree of pathway enrichment, and statistical significance was determined using Fisher’s exact test (*P*<0.05). The KEGG annotations of DEGs were classified according to the type of pathways.

For quantitative real-time PCR (qPCR), we followed the protocol described in Tanveer *et al*. (2024b). The list of primers is given in Supplementary Table S1. For internal reference genes, *C. quinoa* (*Cq) MON1* and *S. oleracea* (*Sp*) *Actin* were used for quinoa and spinach, respectively.

### Statistical analysis, data availability, and visualization

Data were analysed using Statistic v10 software, and the least significant test at the level of *P*≤0.05 was performed to examine the significant effects of ROS treatments on all studied measurements. All figures were made in SigmaPlot v10, and the bars show the SEs. The transcriptomic data generated in this study have been deposited in the National Center for Biotechnology Information (NCBI) Sequence Read Archive (SRA). For *C. quinoa*, the data are available under BioProject accession PRJNA1280883 (SRX29348900, SRX29348899). For *S. oleracea*, the data are available under BioProject accession PRJNA1280894 (SRX29352203, SRX29352202).

## Results

### NaCl triggers growth inhibition and leads to cell-specific H_2_O_2_ accumulation

Both species showed NaCl dose-dependent growth reduction; however, salt stress was less inhibitory for quinoa (Fig. 1A). At the highest salinity level (500 mM), the reduction in DW was 60% for quinoa but 88% for spinach, while at 100 mM NaCl, reduction was 20% and 30% in quinoa and spinach, respectively (Fig. 1A). Na^+^ content in plant roots increased in both species in a dose-dependent manner (Table 1), while K^+^ content progressively declined in spinach but increased in quinoa (Table 1). As a result, quinoa plants had a more optimal K^+^/Na^+^ ratio in roots (Fig. 1C), as well as a higher relative CO_2_ assimilation rate in leaves (Fig. 1B).

**Table 1.** K^+^ and Na^+^ contents (mmol kg⁻^1^ FW) in quinoa and spinach roots under increasing NaCl concentrations

Treatment	Quinoa K^+^	Spinach K^+^	Quinoa Na^+^	Spinach Na^+^
Control	20.08±0.54	28.01±0.70	3.08±0.54	3.19±0.59
100 mM NaCl	27.78±1.04	20.04±0.79	44.68±0.74	89.71±1.24
300 mM NaCl	34.51±1.42	10.13±0.51	82.69±1.52	126.07±2.89
500 mM NaCl	45.938±1.37	4.80±0.56	145.52±2.15	156.06±2.62

Values are means ±SE (*n*=5–6).

We then measured H_2_O_2_ fluorescence intensity in the EZ and MZ of quinoa and spinach roots exposed to 100 mM NaCl (Fig. 1D, E). Both species exhibited elevated ROS compared with their controls, but with distinct spatiotemporal patterns. Spinach showed significantly higher ROS fluorescence in the EZ at all time points, whereas in the MZ, a pronounced difference between species emerged only at 24 h post-treatment (Fig. 1E), most probably as a result of increased expression of the *RBOHA* gene encoding plasma membrane-based NADPH oxidase (Fig. 1F).

### K^+^/Na^+^ fluxes show spatiotemporal specificity in root zones

**Fig. 2.**
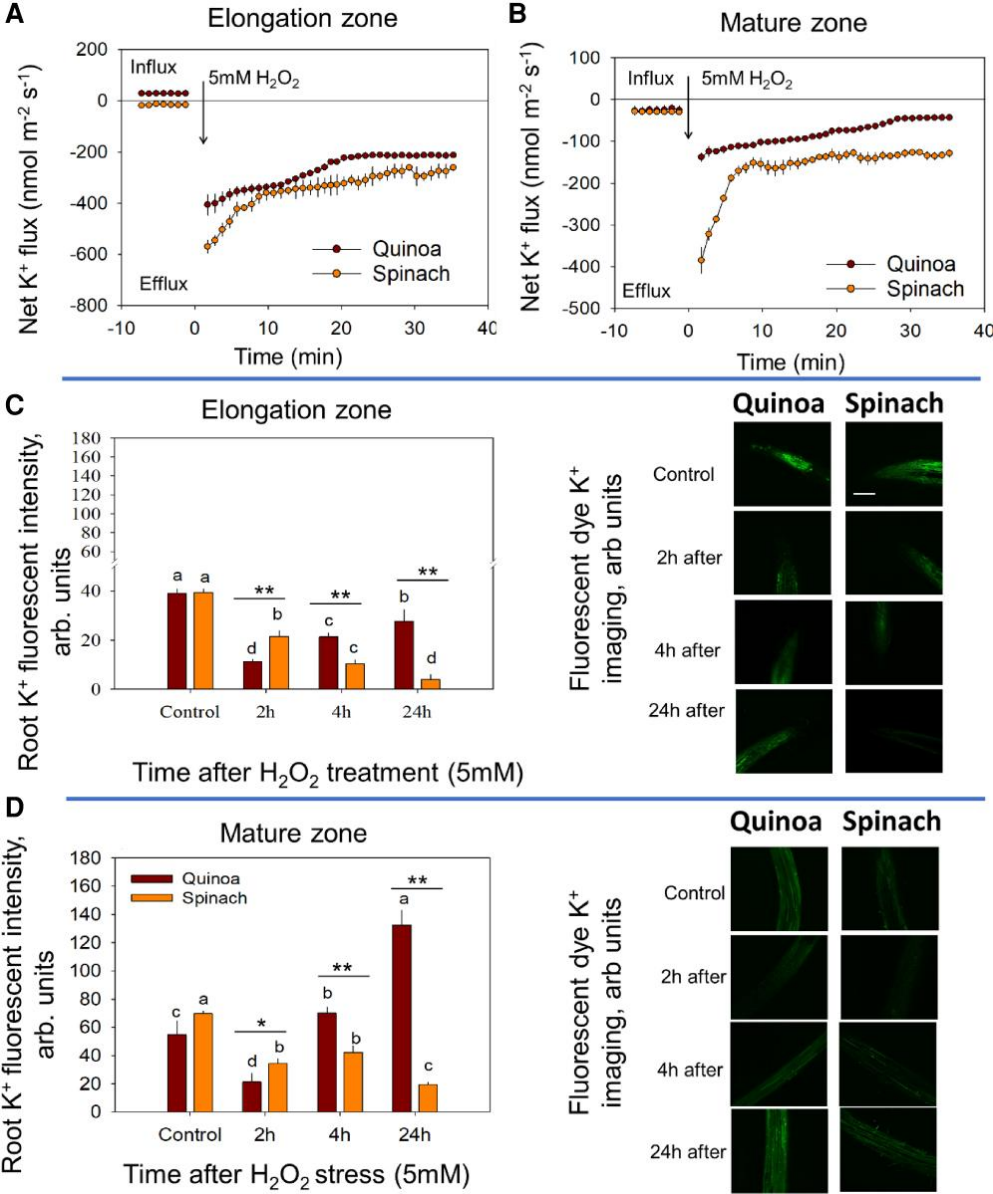
Tissue-specific K^+^ homeostasis under ROS stress. The transient K^+^ flux response from the (A) elongation zone and (B) mature zone in response to ROS stress. The K^+^ fluorescence intensity in the (C) elongation zone and (D) mature zone in response to 5 mM H_2_O_2_ in quinoa and spinach. The root K^+^ fluorescence intensity was calculated using ImageJ software. Data are the mean ±SE [*n*=160–240; 15–30 cells analysed for at least 7–9 individual roots (biological replicates)]. Error bars are SEs, and different letters show that data were significantly different at *P*≤0.05 within each plant species across treatments and time points, while the asterisks show the significant difference between both species at the individual time point of stress at *P≤0.05 and **P≤0.01, respectively.

H_2_O_2_, a key ROS, acts as both an oxidant and signalling molecule in plants under salinity stress (Hossain and Dietz, 2016; Tanveer and Ahmed, 2020). Accordingly, we examined K^+^ and Na^+^ fluxes and their fluorescence intensities in the root EZ and MZ under ROS stress. Acute H_2_O_2_ treatment caused transient K^+^ efflux in both root zones in both species; however, the magnitude of K^+^ efflux was more severe in glycophytic spinach than in quinoa (Fig. 2A, B). The EZ was more ROS sensitive compared with the mature zone.

Profiling time-dependent intracellular K^+^ distribution in different root zones revealed that the EZ of quinoa exhibited a biphasic K^+^ response, with a 60% decrease at 2 h post-H_2_O_2_ treatment followed by its recovery by 24 h (Fig. 2C). In contrast, the EZ of spinach showed a monophasic decline, with continuous K^+^ loss over time under ROS stress (Fig. 2C). Notably, the quinoa MZ retained K^+^ more effectively, displaying a 132% increase in fluorescence intensity by 24 h compared with control (Fig. 2D), whereas K^+^ fluorescence intensity declined in the spinach MZ by 71% (Fig. 2D), suggesting that the halophytic tolerance of quinoa could involve transient K^+^ efflux during ROS burst (2 h) and a subsequent recovery (24 h).

**Fig. 3.**
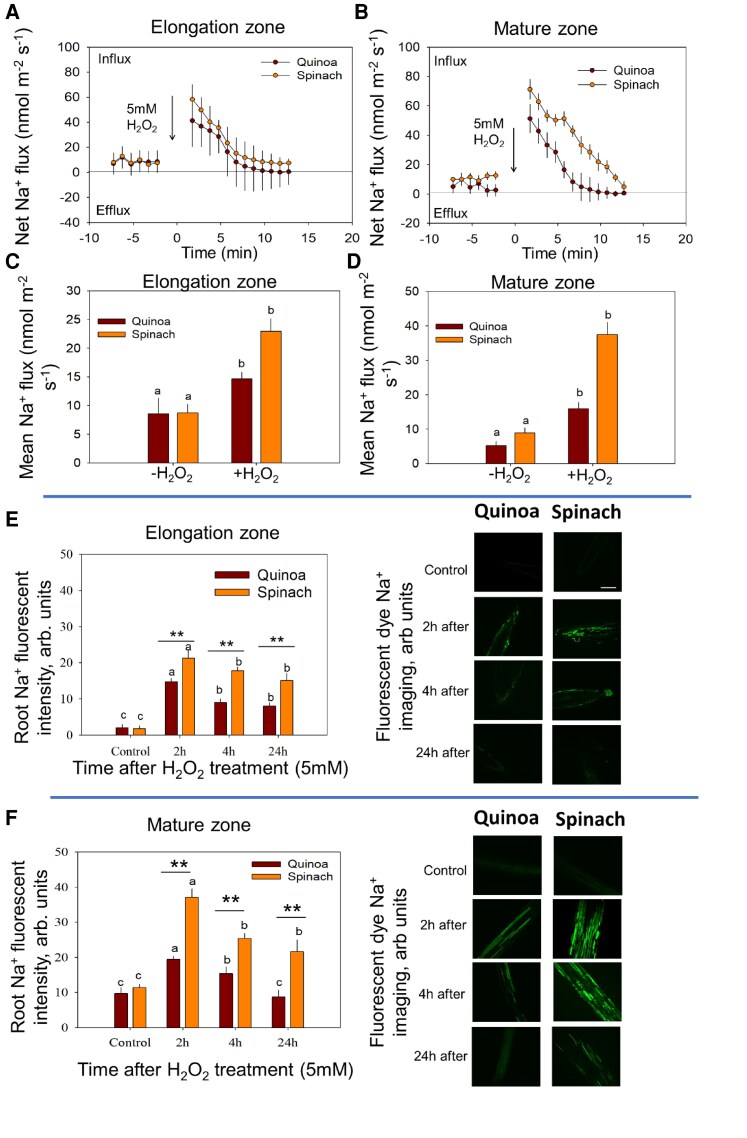
Tissue-specific Na^+^ homeostasis under ROS stress. The transient Na^+^ flux response from the (A) elongation zone and (B) mature zone in response to ROS stress. (C and D) Thee mean Na^+^ flux response from the elongation zone and mature zone, respectively. (E and F) The kinetics of Na^+^ fluorescence intensity in the elongation zone and mature zone in response to ROS in quinoa and spinach, respectively. The root Na^+^ fluorescence intensity was calculated using Image J software. Data are the mean ±SE [*n*=160–240; 15–30 cells analysed for at least 7–9 individual roots (biological replicates)]. Error bars are SEs, and different letters show that data were significantly different at *P*≤0.05 within each plant species across treatments and time points, while the asterisks show the significant difference between both species at the individual time point of stress at *P≤0.05 and **P≤0.01, respectively.

Conversely, ROS triggered transient Na^+^ influx in both zones (Fig. 3A, B). The mean Na^+^ influx was higher in spinach (EZ, 172%; MZ, 267%) than in quinoa (EZ, 58%; MZ, 160%) under ROS compared with control, indicating greater Na^+^ uptake in spinach than quinoa under ROS stress (Fig. 3C, D). The MIFE ion flux data were consistent with CoroNa Green Na^+^ fluorescence dye measurements, showing significantly higher Na^+^ fluorescence intensity in both root zones of spinach plants at all time points under ROS stress (Fig. 3E, F). In contrast, quinoa showed a transiently higher Na^+^ fluorescence intensity (98% and 122% increase in EZ and MZ, respectively, at 2 h), which later declined to near-control levels (Fig. 3E, F).

### Differential tissue specificity of K^+^/Na^+^ was due to subcellular K^+^/Na^+^ redistribution

**Fig. 4.**
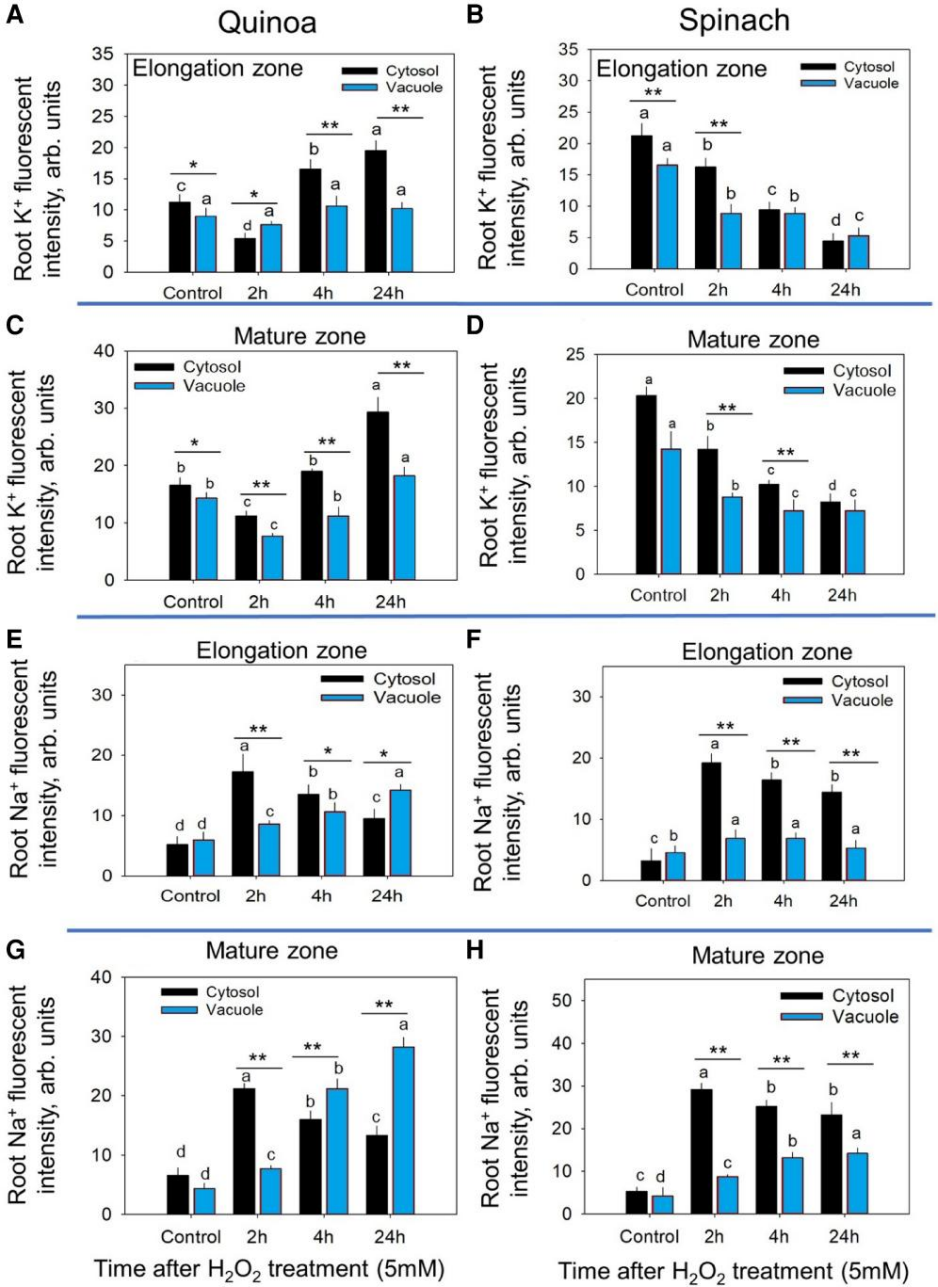
Intracellular distribution of K^+^ and Na^+^ fluorescence intensity in the cytosol and vacuole of quinoa and spinach under ROS stress. (A and B) The K^+^ fluorescence intensity in the elongation zone of quinoa and spinach, respectively. (C and D) The K^+^ fluorescence intensity in the mature zone of quinoa and spinach, respectively, post-ROS stress. (E and F) The Na^+^ fluorescence intensity in the elongation zone of quinoa and spinach, respectively. (G and (H) The Na^+^ fluorescence intensity in the mature zone of quinoa and spinach, respectively, post-ROS stress. The root K^+^ and Na^+^ fluorescence intensity was calculated using Image J software. Data are the mean ±SE [*n*=160–240; 15–30 cells analysed for at least 7–9 individual roots (biological replicates)]. Error bars are SEs, and different letters show that data were significantly different at *P*≤0.05 within each plant species across treatments and time points, while the asterisks show the significant difference between both species at the individual time point of stress at *P≤0.05 and **P≤0.01, respectively.

We then compared the intracellular distributions of K^+^ and Na^+^ in functionally different root zones. In quinoa, cytosolic K^+^ initially decreased (2 h) in the EZ and MZ (Fig. 4A, C) but recovered by 24 h post-ROS stress. At the same time, vacuolar K^+^ remained stable in the EZ of quinoa but followed cytosolic trends in the MZ, reflecting tissue-specific regulation. Conversely, spinach showed progressive cytosolic K^+^ loss in both zones, with vacuolar K^+^ declining significantly by 24 h, indicating systemic K^+^ dysregulation under prolonged oxidative stress (Fig. 4B, D). These results correlated with whole-root K^+^ fluorescence and reinforce that cytosolic K^+^ homeostasis determines overall plant K^+^ status (Shabala, 2017; Wu *et al*., 2018), demonstrating the enhanced capacity of quinoa to employ H_2_O_2_ signalling for K^+^ homeostasis.

The subcellular Na^+^ distribution also revealed species-specific strategies. Quinoa exhibited transient cytosolic Na^+^ accumulation at 2 h post-ROS but achieved effective vacuolar sequestration by 24 h, especially in the MZ (Fig. 4E, G). In contrast, spinach sustained elevated cytosolic Na^+^ with minimal vacuolar partitioning (Fig. 4F, H), demonstrating impaired Na^+^ compartmentalization. Notably, both species showed greater Na^+^-induced cytotoxicity in the EZ than in the MZ, indicating tissue-specific sensitivity and vacuolar Na^+^ sequestration as a critical tolerance mechanism under ROS stress.

### Transcriptomic profiling of reactive oxygen species-responsive differentially expressed genes in quinoa and spinach

We performed transcriptomics analysis in quinoa and spinach roots at 2 h post-stress—a time point with the most critical differences in the cellular K^+^/Na^+^ homeostasis. High-quality RNA-seq data were obtained for both quinoa and spinach, with Q20 and Q30 scores exceeding 98% and 95%, respectively (Supplementary Table S2). The clean reads were mapped to their respective reference genomes, with mapping efficiencies ranging from 92.13–92.42% in control quinoa (q-Control) to 93.08–93.50% in ROS-treated spinach (s-ROS) (Supplementary Table S2). Differential expression analysis (|log2FC|≥1 or ≤ −1, FDR ≤0.05) identified 8658 DEGs in quinoa (3962 up- and 4696 down-regulated) and 3164 DEGs in spinach (1561 up- and 1603 down-regulated) (Supplementary Fig. S1A; Supplementary Tables S3, S4). Volcano and raincloud plots revealed stark contrasts. Quinoa showed right-skewed up-regulation (median log2FC=+2.1) with tight dispersion, indicating coordinated stress gene activation. However, spinach displayed a bimodal distribution (peaks at log2FC±1.8), suggesting concurrent suppression of housekeeping genes and erratic induction of damage control genes (Supplementary Fig. S1B, C).

Furthermore, KEGG pathway analysis underscored species-specific adaptations, with 30 pathways enriched in quinoa (1818 DEGs) compared with 19 in spinach (539 DEGs) (Supplementary Tables S5, S6). Key pathways—MAPK signalling (Ko04016), phytohormone signalling (Ko04075), phenylpropanoid biosynthesis (Ko00940), plant–pathogen interactions (Ko04626), and isoflavonoid biosynthesis (Ko00943)—were significantly and differentially regulated (Supplementary Fig. S1D, E). These findings highlighted robust transcriptional reprogramming and pathway activation of quinoa, contrasting with the less coordinated response of spinach, aligning with their divergent ROS tolerance mechanisms.

### Identification and qPCR of reactive oxygen species-responsive differentially expressed genes in quinoa and spinach

We then focused on DEGs related to K^+^/Na^+^ homeostasis, MAPK signalling, and plant hormone signal transduction pathways. The ROS stress differentially regulated these DEGs relating to K^+^/Na^+^ homeostasis in both species.

#### K^+^ homeostasis

**Fig. 5.**
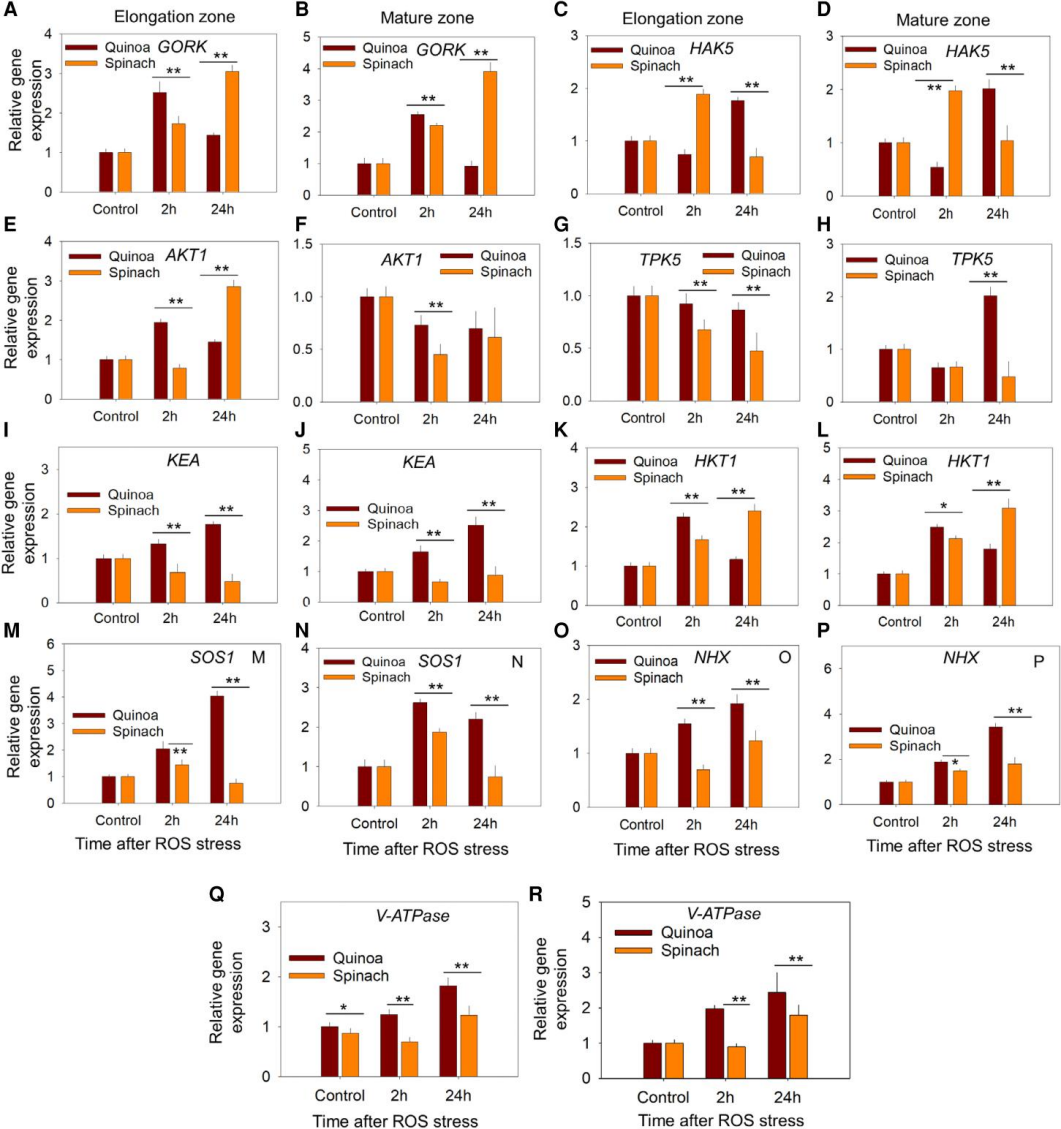
Gene expression of different ion channels and transporters regulating K^+^/Na^+^ homeostasis in the elongation and mature zone of quinoa and spinach roots in response to ROS at 2 h and 24 h. *GORK* (Gated Outward-Rectifying K⁺ channel), *HAK5* (High-Affinity K^+^ transporter 5), *AKT1* (Arabidopsis K^+^ Transporter 1), *TPK5* (Two-Pore K^+^ channel 5), and *KEA* (K^+^ Efflux Antiporter) regulate K^+^ homeostasis, while *HKT1* (High-Affinity K^+^ Transporter 1), *SOS1* (Salt Overly Sensitive 1), *HX* (Na^+^/H⁺ Exchange), and *V-type ATPase* regulate Na^+^ homeostasis. Data are the mean ±SE (*n*=3); error bars are SEs, and the asterisks show the significant difference between both species at the individual time point of stress at *P≤0.05 and **P≤0.01, respectively.

Transcriptomic analysis showed that *AKT1* (Arabidopsis K^+^ Transporter 1) and *KEA* (K^+^-Efflux Antiporter) were up-regultaed, but *KAT3* (K^+^ channel in *Arabidopsis thaliana*), *KUP12* (K^+^ Uptake Permease), *TPK5* (Two-Pore K^+^ channel 5), and *HAK5* (High-Affinity K^+^ transporter 5) were down-regulated in quinoa (Table 2; Supplementary Table S7). In contrast, *KUP12* was down-regulated while *KAT3* and *HAK5* were up-regulaed spinach, with *TPK5* and *KEA* being undetected (Table 2; Supplementary Table S8). These opposing trends reveal species-specific transcriptional adaptations to oxidative stress. The qPCR of the key genes revealed that *GORK* (regulates K^+^ efflux) was up-regulated in both species, but the EZ of quinoa showed higher expression at 2 h, while spinach displayed greater up-regulation in both root zones by 24 h (Fig. 5A, B). *HAK5* expression in quinoa initially decreased (25% in the EZ, 45% in the MZ at 2 h) but later increased by 76% in the EZ and 101% in the MZ at 24 h, whereas spinach exhibited an early increase (89% in the EZ, 97% in the MZ at 2 h) followed by a decline (30%) in the EZ only (Fig. 5C, D). *AKT1* expression in the EZ of quinoa increased steadily (95% at 2 h, 44% at 24 h), while spinach showed initial suppression (22% at 2 h) before a sharp rise (185% at 24 h) (Fig. 5E). In the MZ, *AKT1* expression decreased in both species post-ROS stress, with a sharp decline in its expression at 2 h post-ROS stress (Fig. 5F). *TPK5* (responsible for K^+^ vacuolar buffering) decreased in the EZ of both species but increased by 101% in the MZ of quinoa at 24 h only (Fig. 5G, H). Conversely, *KEA* expression was significantly increased in the EZ and MZ of quinoa, whereas it was down-regulated in spinach post-ROS stress (Fig. 5I, J).

**Table 2.** Regulation of selected DEGs relating to MAPK signalling, hormone signalling, and K^+^/Na^+^ homeostasis in quinoa and spinach roots at 2 h under ROS stress

DEGs	Quinoa	Spinach	Annotation
	Gene ID (log2FC) status	Gene ID (log2FC) status	
MAPK signalling			
*MAPKKK20*	gene26283 (2.53) up	SOV3g042580 (−1.93) down	Mitogen-activated protein kinase kinase kinase 20
*BOHA*	gene43032 (−1.39) down	NewGene_251 (3.75) up	Respiratory burst oxidase homologue protein A-like
*RBOHB-like*	gene50219 (1.35) up	Not detected	Respiratory burst oxidase homologue protein B-like
*RBOHH*	Not detected	SOV6g026410 (1.26) up	Respiratory burst oxidase homologue protein H
*MAPKKK17*	gene27310 (2.41) up	SOV1g041060 (1.295) up	Mitogen-activated protein kinase kinase kinase 17
*MAPK2*	gene6920 (−1.55) down	SOV6g039650 (−1.49) down	Mitogen-activated protein kinase homologue MMK2-like
Ethylene signalling			
*ACO*	gene39885 (1.51) up	SOV3g008510 (−1.09) down	1-Aminocyclopropane-1-carboxylate oxidase
*EBF1*	gene12258 (−1.32) down	Not detected	EIN3-binding F-box protein 1-like
*ERF1B*	gene19123 (2.01) up	SOV5g001510 (−1.59) down	Ethylene-responsive transcription factor 1B
*ERF3*	gene4669 (−2.19) down	SOV1g026000 (−2.57) down	Ethylene-responsive transcription factor ERF003
*ERF5*	gene43205 (−1.03) down	SOV4g002590 (3.05) up	Ethylene-responsive transcription factor 5-like
*ERF8*	Not detected	SOV4g040740 (−1.14) down	Ethylene-responsive transcription factor 8
*EIL5*	gene38530 (−1.24) down	Not detected	Putative ETHYLENE INSENSITIVE 3-like 4 protein
*PLT2*	gene21709 (2.45) up	SOV4g001160 (1.80) up	AP2-like ethylene-responsive transcription factor PLT2
ABA signalling			
*PYL2*	gene56056 (1.05) up	SOV1g035460 (2.91) up	Abscisic acid receptor PYL2
*PYL4*	gene41705 (1.77) up	SOV6g038130 (4.46) up	Abscisic acid receptor PYL2
*ABI5*	gene38455 (−2.95) down	Not detected	Protein ABSCISIC ACID-INSENSITIVE 5
*ASR1*	gene21439 (−3.12) down	SOV2g024680 (1.82) up	Abscisic stress-ripening protein 1
*CYP707A4*	gene44259 (−2.64) down	SOV2g024760 (−1.72) down	Abscisic acid 8'-hydroxylase 4
Potassium			
*AKT1*	gene18266 (1.08) up	Not detected	Potassium channel AKT1-like
*KAT3*	NewGene_7269 (−1.58) down	SOV6g029750 (1.35) up	Potassium channel KAT3
*KUP12*	gene36982 (−1.06) down	SOV1g039340 (−1.18) down	Potassium transporter 12
*TPK5*	gene18927 (−1.24) down	Not detected	Two-pore potassium channel 5-like
*HAK5*	gene51504 (−2.31) down	SOV1g033390 (5.13) up	Potassium transporter 5-like
*KEA*	gene3111 (1.07) up	Not detected	K^+^ efflux antiporter 5
Sodium			
*HKT1*	gene2286 (1.73) up	SOV2g010820 (1.69) up	Sodium transporter HKT1-like
*NHX2*	gene50028 (−1.28) down	Not detected	Vacuolar sodium/hydrogen exchanger 2
*V-ATPase*	NewGene_3070 (2.74) up	SOV1g011140 (−7.43) down	V-type proton ATPase

#### Na^+^ homeostasis

The transcriptomics analysis revealed that *HKT1* was up-regulated in both species, but *NHX* was only down-regulated in quinoa (undetected in spinach) (Table 2). Notably, vacuolar *V-ATPase* was up-regulated in quinoa but down-regulated in spinach, indicating divergent Na^+^ regulation strategies under oxidative stress (Table 2). The qPCR of genes relating to Na^+^ homeostasis showed that *HKT1* expression was transiently induced in both species at 2 h (quinoa, 125% EZ, 112% MZ; spinach, 67% EZ, 149% MZ) but diverged dramatically by 24 h, with only quinoa showing suppression of expression while spinach showed extreme up-regulation (140% EZ; 210% MZ) (Fig. 5K, L). Quinoa effectively countered Na^+^ stress through sustained up-regulation of *SOS1* (175% EZ; 120% MZ at 24 h, Fig. 5M, N) and *NHX* (92% EZ; 195% MZ at 24 h, Fig. 5O, P). In contrast, spinach displayed reduced SOS1 expression (72% EZ, 66% MZ) and weaker *NHX* induction (35% EZ, 38% MZ) compared with quinoa at 24 h post-ROS treatment (Fig. 5M–P), revealing a less efficient *SOS1*–*NHX* regulatory mechanism for cytosolic Na^+^ control under ROS stress. Further, the expression of *V-ATPase* was increased in both species under ROS; however, quinoa showed relatively higher expression than spinach (Fig. 5Q, R). These contrasting transcriptional responses further highlight the superior K^+^/Na^+^ homeostasis regulation of quinoa compared with spinach under ROS stress.

#### Mitogen-activated protein kinase signalling

**Fig. 6.**
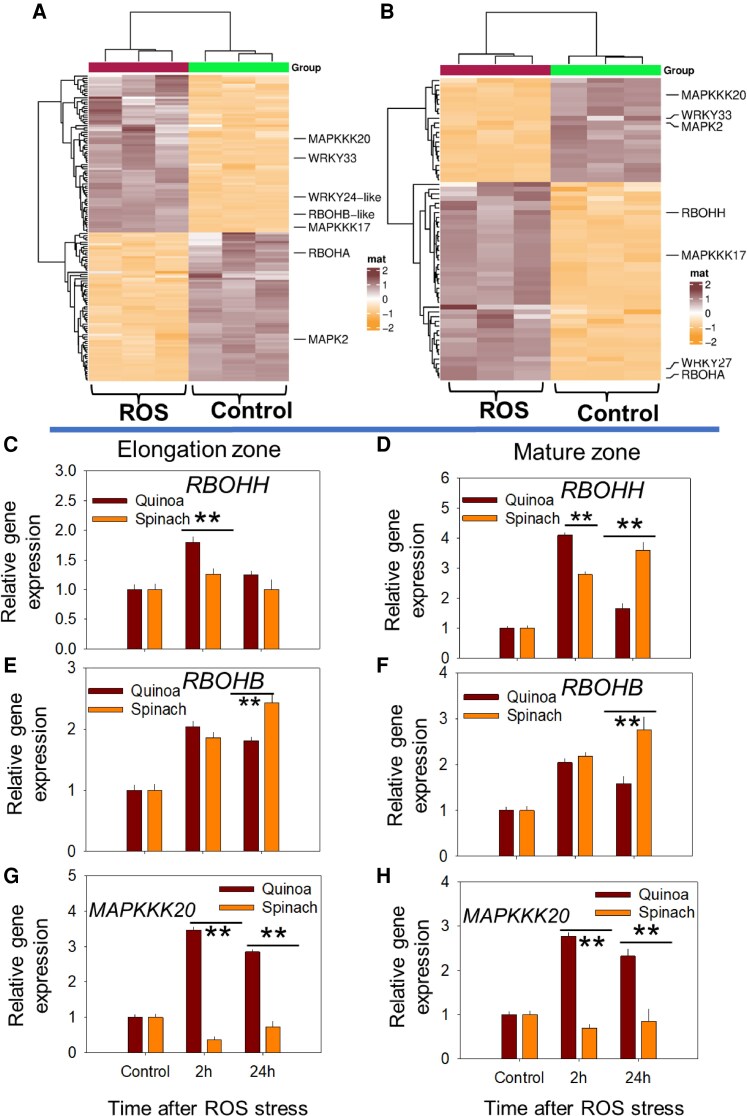
Overview of DEGs in roots of quinoa and spinach under ROS stress. Heatmap of DEGs related to MAPK signalling in the roots of (A) quinoa and (B) spinach at 2 h under ROS stress. qPCR of (C and D) *RBOHH* (Respiratory Burst Oxidase Homologue H), (E and F) *RBOHB* (Respiratory Burst Oxidase Homologue B), and (G and H) *MAPKKK20* (Mitogen-Activated Protein Kinase Kinase Kinase 20) in the elongation zone and mature zone at 2 h and 24 h post-ROS stress. Data are the mean ±SE (*n*=3); error bars are SEs, and the asterisks show the significant difference between both species at the individual time point of stress at *P≤0.05 and **P≤0.01, respectively.

The hierarchical clustering analysis (heatmap) revealed distinct ROS-responsive expression patterns in both species relating to MAPK signalling (Fig. 6A, B), with quinoa showing stronger activation (76 up-regulated and 72 down-regulated DEGs in MAPK signalling, Supplementary Table S9) compared with spinach (45 up-regulated and 23 down-regulated DEGs, Supplementary Table S10). Notably, several DEGs displayed opposite types of regulation between species: genes such as *RBOHA* and *WRKY28* were down-regulated in quinoa but up-regulated in spinach, while others showed species-specific regulation under ROS stress (Table 2), including quinoa-specific DEGs (*RBOHB*, *PP2C*, *WRKY57*, and *WRKY40*) and spinach-specific DEGs (*RBOHH*, *WRKY53*, and *MAPKKK5*). Some DEGs were up-regulated in quinoa but down-regulated in spinach, including *MAPKKK20* under ROS stress (Table 2).

qPCR data showed that *RBOHH* remained consistently higher in the EZ of quinoa, though its expression in the MZ declined 59% in quinoa by 24 h, while spinach showed increased expression by 75%, compared with 2 h post-ROS stress (Fig. 6C, D). The *RBOHB* expression showed early up-regulation in the EZ of quinoa (34% higher than spinach at 2 h), while spinach surpassed quinoa by 61% at 24 h (Fig. 6E, F). Notably, *MAPKKK20* was strongly induced in quinoa but unresponsive in spinach (Fig. 6G, H), demonstrating the more robust MAPK pathway activation of quinoa under oxidative stress.

#### Plant hormonal signalling

**Fig. 7.**
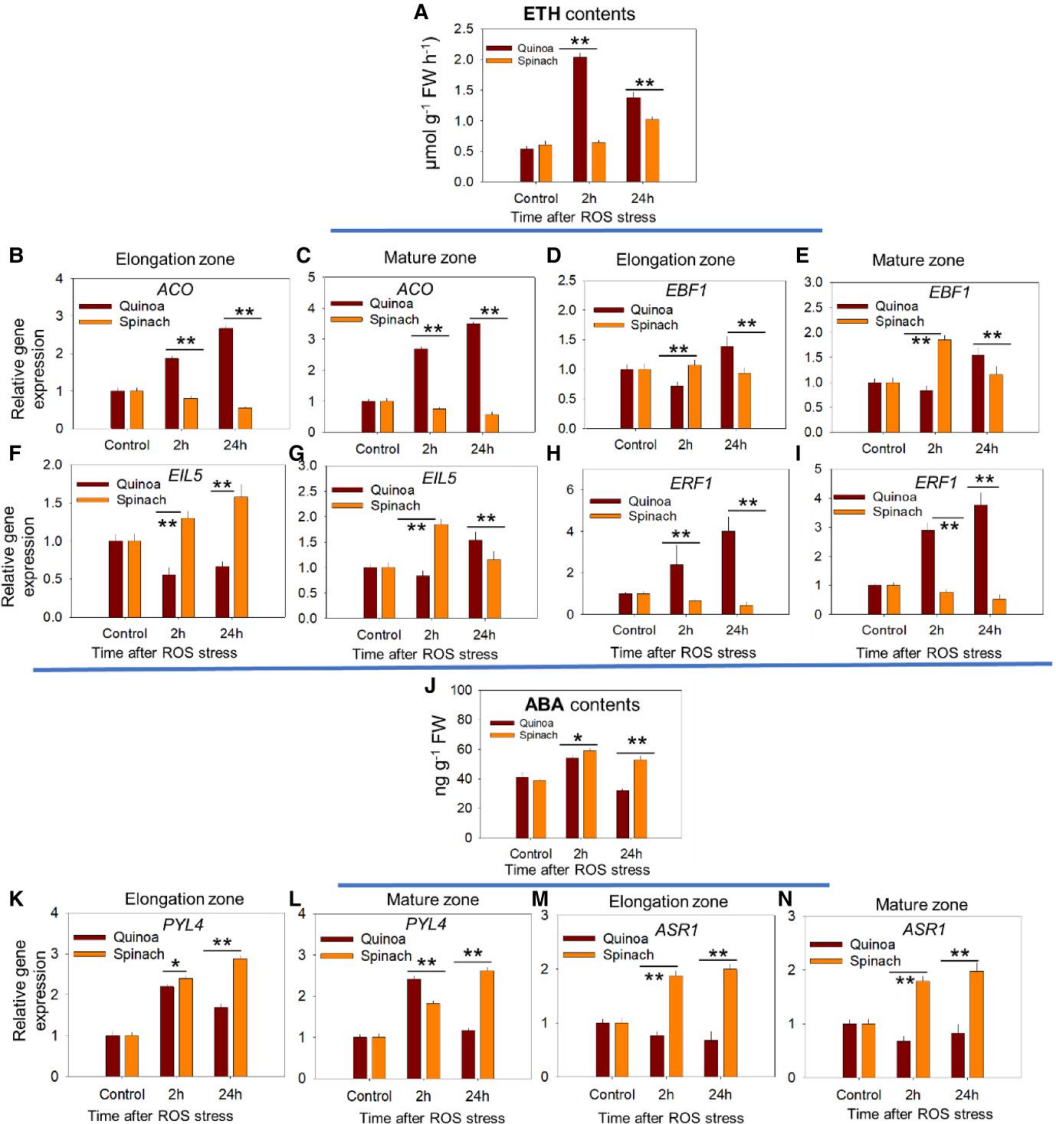
The tissue-specific hormonal response of quinoa and spinach to ROS stress. (A) Endogenous ethylene (ETH) contents in roots of quinoa and spinach at 2 h and 24 h post-ROS stress. qPCR of genes related to ETH signalling: (B and C) *ACO* (1-aminocyclopropane-1-carboxylic acid oxidase), (D and E) *EBF1* (EIN3-Binding F-Box protein 1), (F and G) *EIL5* (EIN3-Like 5), and (H and I) *ERF1* (Ethylene Response Factor 1), in the elongation zone and mature zone of quinoa and spinach under ROS stress, respectively. (J) Endogenous ABA contents in roots of quinoa and spinach at 2 h and 24 h post-ROS stress. qPCR of genes related to ABA signalling: (K and L) *PYL4* (Pyrabactin Resistance 1-Like 4) and (M and N) *ASR1* (ABA Ripening-induced Protein 1), in the elongation zone and mature zone, respectively, under ROS stress. The K^+^/Na^+^ fluorescence intensity in the (O and P) ETH- and (Q and R) ABA-pre-treated elongation and mature zone of both species at 2 h and 24 h post-ROS stress, respectively. Data are the mean ±SE (*n*=3). Error bars are SEs, and the asterisks show the significant difference between both species at the individual time point of stress at *P≤0.05 and **P≤0.01, respectively.

The analysis of phytohormone-related DEGs revealed striking species-specific regulation under ROS stress (Supplementary Fig. S2; Supplementary Tables S7, S8). Given the stress-regulatory role of ETH and ABA in plants under salt stress, the heat map analysis revealed distinct responsiveness of ETH- and ABA-related DEGs to ROS in both species (Supplementary Fig. S2). Key ETH pathway genes—*1-aminocyclopropane-1-carboxylate oxidase* (*ACO*), ETH-responsive transcription factor genes, and ETH signalling regulators [*EIN3-binding F-box protein 1* (*EBF1*) and *ETHYLENE INSENSITIVE 3-like 5* (*EIL5*)]—showed contrasting responses: *ACO* and *ERF1* were up-regulated quinoa while they were down-regulated in spinach (Table 2). The *ERF5/8* DEGs were responsive to ROS in spinach only, while *EBF1/EIL5* DEGs were responsive to ROS only in quinoa (Table 2). Furthermore, at all time points post-ROS stress, quinoa roots accumulated more ETH than spinach (Fig. 7A), consistent with higher expression of *ACO* in both root zones but decreased expression in spinach (reduction of 41% in the EZ and 43% in the MZ at 24 h) (Fig. 7B, C). *EBF1* exhibited temporal regulation—higher expression at 2 h in spinach (∼30% in both zones) but decreased in quinoa (45% EZ, 14% MZ), whereas at 24 h, this pattern reversed, with spinach declining (45% EZ, 67% MZ) and quinoa increasing (34% EZ, 95% MZ) its expression compared with 2 h post-ROS stress (Fig. 7D, E). EIL5 expression was higher in spinach but declined in quinoa at 2 h in both root zones, with an opposite trend at 24 h in the MZ of both species post-ROS stress (Fig. 7F, G). *ERF1* expression remained consistently higher in quinoa than in spinach at all time points post-ROS stress (Fig. 7H, I).

Key ABA-related DEGs were also differentially responsive to ROS (Supplementary Tables S7, S8). While the ABA receptor gene *Pyrabactin Resistance Like 4* (*PYL4*) was up-regulated in both species, *ABA insensitive 5* (*ABI5*) and *Abscisic stress ripening protein 1* (*ASR1*) were down-regulated in quinoa, whereas *ASR1* was up-regulated in spinach but *ABI5* was not detectable (Table 2). Further, ABA preferentially accumulated in spinach roots, while quinoa showed reduced ABA accumulation (21%) by 24 h post-ROS (Fig. 7J) and the qPCR data showed that *PYL4* and *ASR1* were more highly expressed in spinach, particularly at 24 h, whereas quinoa showed a progressive decline in *ASR1* in both root zones (Fig. 7K–N). These results highlight species-specific regulatory strategies in stress-responsive hormone signalling.

### Phytohormone–ion–antioxidant crosstalk under reactive oxygen species stress

**Fig. 8.**
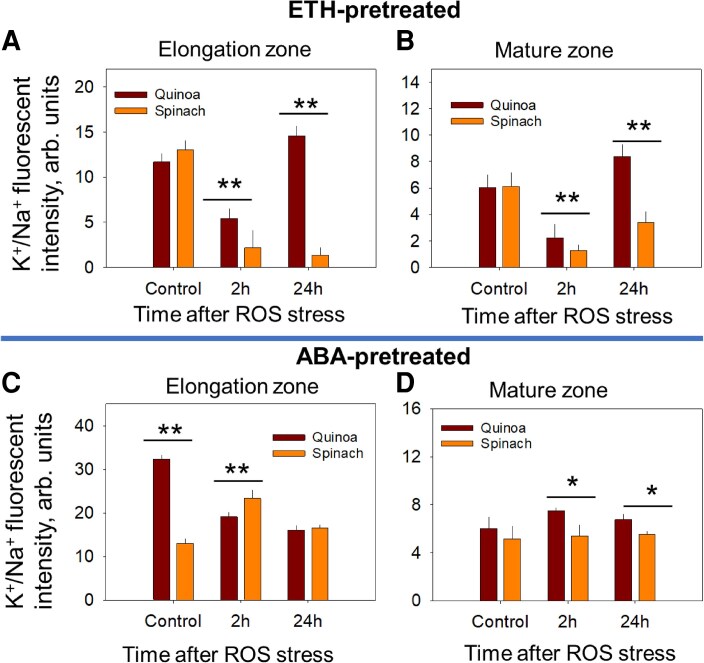
The K^+^/Na^+^ fluorescence intensity in the ethylene (ETH)- and ABA-pre-treated quinoa and spinach roots under ROS stress. (A) and (B) The K^+^/Na^+^ fluorescence intensity in the ETH-pre-treated elongation zone and the mature zone at 2 h and 24 h post-ROS stress, respectively. (C) and (D) The K^+^/Na^+^ fluorescence intensity in the ETH-pre-treated elongation zone and the mature zone at 2 h and 24 h post-ROS stress, respectively. Data are the mean ±SE [*n*=160–240; 15–30 cells analysed for at least 7–9 individual roots (biological replicates)]. Error bars are SEs, and the asterisks show the significant difference between both species at the individual time point of stress at *P≤0.05 and **P≤0.01, respectively.

We then looked at the effect of exogenously applied ETH and ABA on K^+^/Na^+^ homeostasis in ROS-exposed roots. An ∼27% higher K^+^/Na^+^ fluorescence intensity (K^+^-to-Na^+^ fluorescence intensity ratio) was observed in ETH-pre-treated root zones of quinoa by 24 h post-ROS stress (Fig. 8A, B), while spinach showed a significant decline in K^+^/Na^+^ fluorescence intensity in both root zones post-ROS stress (Fig. 8A, B). Conversely, ABA pre-treatment increased K^+^/Na^+^ in the EZ of spinach only at 2 h post-ROS stress (Fig. 8C), while it minimally influenced K^+^/Na^+^ fluorescence intensity in the MZ in both species (Fig. 8D).

**Fig. 9.**
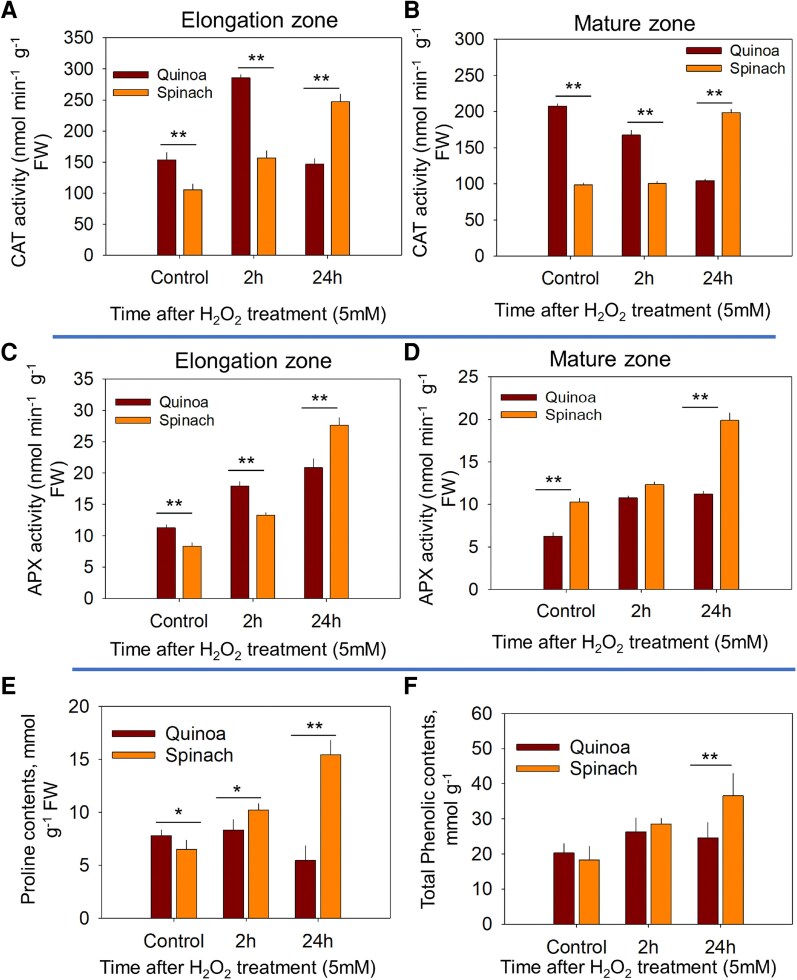
The regulation of antioxidant activities of catalase (CAT) and ascorbate peroxidase (APX) in the elongation zone and mature zone of quinoa and spinach post-ROS stress. (A) and (B) CAT and APX activities in the elongation zone, (C) and (D) CAT and APX activities in the mature zone of both species at 2 h and 24 h post-ROS stress, respectively. (E) and (F) Proline and total phenolic contents in roots at 2 h and 24 h post-ROS stress, respectively. Data are the mean ±SE (*n*=6). Error bars are SEs, and the asterisks show the significant difference between both species at the individual time point of stress at *P≤0.05 and **P≤0.01, respectively.

To explore a possible causal link between hormonal signalling and ROS accumulation, we then looked at the activity of two antioxidant enzymes involved in H_2_O_2_ scavenging, namely APX CAT. A transient CAT activation (peaking at 2 h) was observed in the quinoa EZ, while spinach showed higher activities at 24 h post-ROS stress in both root zones (Fig. 9A, B). APX activity increased over time in both species, with quinoa showing higher activity at 2 h, while spinach surpassed it by 24 h post-ROS stress in both root zones, indicating prolonged antioxidant up-regulation in spinach (Fig. 9C, D). Moreover, proline and total phenolic contents were also higher in spinach roots as compared with quinoa at 24 h post-ROS treatment (Fig. 9E, F).

## Discussion

### Tissue-specific reactive oxygen species-sensing hub for K^+^ regulation

Salinity stress induces cytotoxic oxidative stress as a secondary effect, which disrupts ion homeostasis in plants (Isayenkov and Maathuis, 2019). Glycophytes demonstrated a 2.5- to 3-fold increase in ROS accumulation in roots under NaCl stress (Demidchik *et al*., 2010; AbdElgawad *et al*., 2016; Chakraborty *et al*., 2016), resulting in activation of ROS-sensitive *GORK* channels, mediating K^+^ efflux and potentially exacerbating K^+^ deficiency (Demidchik, 2014; Tanveer *et al*., 2024a). However, as different root tissues play distinct functional roles— the metabolically active EZ and the nutrition-focused MZ (Verbelen *et al*., 2006; Baluška *et al*., 2010; Gao *et al*., 2015; Gill *et al*., 2018)—ROS-mediated K^+^ regulation must also operate in a tissue-specific manner. To directly dissect such a ROS signalling module, we employed exogenous H_2_O_2_ as ROS treatment, isolating its spatiotemporal role in K^+^/Na^+^ homeostasis from the complex ionic effects of NaCl. Our results showed that the EZ (at least in halophytic quinoa) probably serves as a critical hub for early ROS perception and K^+^ flux modulation. Despite the higher dry biomass of quinoa compared with that of spinach under NaCl stress (Fig. 1A), its EZ (based on K^+^ fluorescence intensity) displayed a rapid yet transient K^+^ efflux (2 h post-ROS), followed by recovery (24 h post-ROS), indicative of dynamic K^+^ signalling that prevents prolonged K^+^ depletion (Fig. 2C). In contrast, glycophytic spinach exhibited a monophasic K^+^ decline in the EZ and MZ post-ROS stress (Fig. 2C, D), reflecting an inability to restore K^+^ homeostasis, which may contribute to its salinity sensitivity. This spatial specificity suggests tissue- and species-specific temporal regulation of K^+^ efflux under ROS stress, consistent with a previous report on rice, where higher GORK expression in the root apex was observed compared with mature zones (J. Liu *et al*., 2019). This further aligns with the established role of the EZ in stress sensing (Wu *et al*., 2015b), where ROS act as signalling molecules to modulate ion channels such as *GORK* and *NSCC*. Moreover, the early activation (2 h) of ROS accumulation and K^+^ efflux in quinoa can also be ascribed to the signalling role of K^+^ efflux (Shabala, 2017), which may shift cellular metabolism towards defence mechanisms (Demidchik, 2014) or initiating systemic root-to-shoot signalling (S. Shabala *et al*., 2016).

Since *RBOHB* and *RBOHH* expression peaked earlier (2 h post-stress) in quinoa compared with spinach (24 h post-stress) (Fig. 6C–F), the biphasic K^+^ response in quinoa could also arise from fewer ROS-sensitive *GORK* channels (Wang *et al*., 2017; Adem *et al*., 2020) or enhanced antioxidant activity. However, since antioxidant enzyme activity was higher in spinach under long-term (24 h) ROS stress (Fig. 9), the latter explanation seems less plausible. Instead, quinoa may possess intrinsic mechanisms that may reduce ROS sensitivity, enabling precise control over K^+^ fluxes. In this context, our study showed the tissue-specific regulation of K^+^ flux responses at the post-translational level following ROS stress. In quinoa, a transient up-regulation of *GORK* expression occurred in both root zones at 2 h post-ROS stress compared with spinach (Fig. 5A, B), coinciding with an initial decline in K^+^ fluorescence intensity (Fig. 2C, D). At 24 h post-ROS stress, *GORK* activation was significantly reduced, corresponding to a recovery in K^+^ fluorescence intensity. In contrast, spinach showed 2-fold higher *GORK* expression in spinach at 24 h post-ROS stress than quinoa (Fig. 5A, B). The physiological significance of ROS-induced *GORK* expression may also lie in its role as a ‘metabolic switch’, where controlled K^+^ efflux temporarily inhibits energy-consuming biosynthetic processes, redirecting resources towards defence and repair mechanisms (Demidchik, 2014; Shabala, 2017; Adem *et al*., 2020; Rubio *et al*., 2020). However, this signalling function must be carefully balanced against the nutritional K^+^ requirements of the plant. Our data reveal two complementary mechanisms that could enable quinoa to resolve this dilemma: first, the transient nature of ROS-induced *GORK* up-regulation in parallel with ROS dynamics (Fig. 5A, B); and second, the coordinated induction of *AKT1* transcripts at 2 h (Fig. 5E, F) followed by *HAK5* up-regulation at 24 h (Fig. 5C, D), resulting in a 3-fold higher K^+^ fluorescence intensity compared with controls (Fig. 2D). Thus, a ‘compensation mechanism’ appears to exist in quinoa, which we previously suggested in the leaf mesophyll of quinoa (Tanveer *et al*., 2024b), that might allow quinoa to utilize K^+^ efflux for signalling purposes while maintaining adequate cellular K^+^ levels. The efficiency of this dual-phase response—involving both rapid channel activation and subsequent transporter induction—appears crucial for salinity tolerance (L. Shabala *et al*., 2016).

Other evidence also supports such distinct spatiotemporal patterns of intracellular K^+^ homeostasis between species under ROS stress. In quinoa, both root zones exhibited rapid cytosolic K^+^ depletion at 2 h, followed by complete recovery to levels exceeding controls by 24 h (Fig. 4A, C). While vacuolar K^+^ remained stable in the EZ (Fig. 4A), the MZ vacuoles mirrored the cytosolic K^+^ fluorescence intensity patterns (Fig. 4C). In contrast, spinach displayed progressive declines in both cytosolic and vacuolar K^+^ pools across both zones (Fig. 4B, D), aligning with its impaired K^+^ retention in both root zones (Fig. 2), indicating that species-specific strategies could be responsible for maintaining K^+^ homeostasis during oxidative challenge. In this context, our transcriptomics and qPCR results showed that in quinoa, *TPK5* expression was down-regulated in the EZ (Fig. 5G), while it increased by 2-fold in the MZ at 24 h (Fig. 5H), and *KEA* expression was increased in both root zones of quinoa (Fig. 5I, J) (unlike spinach, where it was suppressed). This spatiotemporal control aligns with studies showing that ROS modulate *TPK* gating (Isayenkov and Maathuis, 2019), while *KEA* activity is critical for stress-induced K^+^ buffering (Tsujii *et al*., 2019). Crucially, the ability of quinoa to transiently release K^+^ from the vacuole (via *TPK5*) without its net loss from the cell—leveraging *KEA* for reuptake mirrors its ROS-inducible HAK5/AKT1 induction (Fig. 5C–F), thereby creating a closed-loop system for K^+^ redistribution. In contrast, the concurrent *TPK5* suppression and *KEA* down-regulation (Fig. 5G–J) in spinach probably led to uncontrolled K^+^ depletion, underscoring how ROS–*TPK5*–*KEA* crosstalk determines stress resilience. These findings extend prior models (Demidchik, 2014; L. Shabala *et al*., 2016; Shabala, 2017; J. Liu *et al*., 2019) by demonstrating that tissue specificity of transporter expression—not just activity—underlies efficient K^+^ management under stress.

### Tissue-specific Na^+^ handling could underlie halophytic tolerance in quinoa

Plants maintain cytosolic Na^+^ balance under salinity through Na^+^ exclusion and/or vacuolar sequestration, with varying efficiency across root zones based on stress sensitivity (L. Shabala *et al*., 2016; Wu *et al*., 2018). Halophytic quinoa and glycophytic spinach may differ in these mechanisms, influenced by tissue-specific ROS signalling and Na^+^ homeostasis. Our results demonstrated that both quinoa and spinach roots showed immediate Na^+^ influx under ROS stress (Fig. 3A, B), but quinoa maintained significantly lower Na^+^ levels in both root zones than spinach (Fig. 3E, F). Further, quinoa exhibited dynamic Na^+^ partitioning—initially higher cytosolic Na^+^ at 2 h shifted to vacuolar accumulation by 24 h (Fig. 4E, G), probably restoring near-basal cytosolic levels. In contrast, spinach retained excessive cytosolic Na^+^ in both zones (Fig. 4F, H), with only slow vacuolar sequestration in the MZ (Fig. 4H), indicating inefficient Na^+^ compartmentalization. These results highlighted the superior ROS tolerance of quinoa through (i) tissue-specific Na^+^ redistribution and (ii) rapid vacuolar sequestration, minimizing Na^+^ cytosolic toxicity.

Several physiological mechanisms may contribute to our observations. Firstly, quinoa exhibited sustained up-regulation of both *SOS1* (Na^+^ exclusion) and *NHX* (vacuolar sequestration) genes across root zones. This dual mechanism allows plants to minimize cytosolic Na^+^ toxicity while maintaining cellular turgor. At the same time, spinach showed only transient activation—*SOS1* at 2 h and *NHX* at 24 h—with significantly lower expression levels than quinoa (Fig. 5M–P). Although *HKT1* was up-regulated in both species, it was suppressed in quinoa by 24 h, while it was overexpressed in spinach (Fig. 5K, L)—suggesting that the persistent Na^+^ influx in spinach reflects a failure to activate compensatory mechanisms. Further, these molecular patterns directly correlate with our Na^+^ fluorescence data in the root EZ and MZ (Fig. 3E, F) and previously established models of *SOS1*-mediated Na^+^ extrusion in root apices (Shi *et al*., 2000) and *NHX*-dependent vacuolar compartmentalization (Wu *et al*., 2015b).

Secondly, our transcriptomic and qPCR analyses revealed key differences in *V-ATPase* gene expression between species. *V-ATPase* energizes ion homeostasis by regulating Na^+^ compartmentalization and K^+^ retention via H^+^ gradients that drive NHX-mediated vacuolar sequestration and *SOS1*-dependent Na^+^ exclusion (Lv *et al*., 2017; Zhou *et al*., 2020). Quinoa exhibited sustained *V-ATPase* up-regulation in both root zones under ROS stress, whereas spinach showed delayed induction (24 h) only in the MZ (Fig. 5Q, R; Table 2). This robust *V-ATPase* activation in quinoa probably fuels (i) *NHX*-dependent Na^+^ sequestration and (ii) *SOS1*-driven Na^+^ extrusion, enabling efficient Na^+^ partitioning. The findings align with reports in halophytes such as *Nitraria sibirica*, where tissue-specific maintenance of K^+^/Na^+^ ratios correlated with enhanced *V-ATPase* and *NHX* activity (Tang *et al*., 2021). In contrast, the weak *V-ATPase* response of spinach correlates with persistent cytosolic Na^+^ accumulation in both root zones after ROS stress (Fig. 3E, F). Thus, the halophytic adaptation of quinoa hinges on rapid, system-wide *V-ATPase* activation—a critical mechanism less efficient in glycophytic spinach under ROS.

Thirdly, the transcriptional zonation of Na^+^ homeostasis aligns with ROS gradients across root zones. For instance, the EZ, with higher ROS levels, showed a preferential *SOS1* up-regulation (2-fold) for Na^+^ exclusion, while the MZ, with lower ROS, displayed dominant NHX induction (1.8-fold) for vacuolar sequestration (Fig. 5). This tissue specificity aligns with the findings in rice, where salt stress induced stronger *OsSOS1* expression in the EZ (20-fold) versus the MZ (6-fold) (Y. Liu *et al*., 2019), indicating the tissue-specific role of H_2_O_2_ in Na^+^ exclusion (Niu *et al*., 2018). In contrast, spinach failed to coordinate transporter expression, maintaining higher *HKT1* expression at 24 h (Fig. 5K, L) and net Na^+^ influx (Fig. 3C, D), resulting in higher Na^+^ fluorescence intensities (Fig. 3E, F). Such transcriptional zonation underscores the precision of the adaptive responses of quinoa, where ROS gradients are exploited to spatially optimize ion transport.

### Tissue-specific hormonal regulation contributes to superior halophytic tolerance

Though both species regulate K^+^/Na^+^ in a tissue-specific manner via regulating ROS signalling, our results also revealed a fundamental dichotomy in how halophytic quinoa and glycophytic spinach are likely to employ hormonal signalling to coordinate tissue-specific ion homeostasis under ROS stress. For instance, the resilience of quinoa could originate from sustained ETH signalling in both root zones, which correlates strongly with its efficient K^+^ retention (Fig. 2) and higher Na^+^ exclusion/vacuolar sequestration (Fig. 5M, P)—a strategy mechanistically linked to ETH-mediated activation of *SOS1* and *NHX* transporter genes (Riyazuddin *et al*., 2020). Conversely, spinach showed higher ABA accumulation with less efficient *SOS1* and *NHX* activation (Fig. 5M–P, J). This aligns with findings in several plant species, where ETH enhances salt tolerance by reinforcing *SOS1* activity (Quan *et al*., 2017; Vaseva *et al*., 2021) while antagonizing the suppressive effects of ABA (Chen *et al*., 2021; Fatma *et al*., 2021).

In the light of that, the broader implications of ROS versus hormonal crosstalk regulating cellular K^+^/Na^+^ are 3-fold. Firstly, halophytes such as quinoa could exploit ETH as an ‘ionic controller’, synchronizing transporter activities spatially with tissue-specific ROS gradients (e.g. *SOS1* dominance in the high-ROS EZ versus *NHX* preference in the low-ROS MZ), indicating tissue-specific ROS–*SOS1* coupling. Glycophytes such as spinach default to ABA-mediated osmotic adjustments and higher antioxidants—a metabolically costly but evolutionarily constrained strategy (Li *et al*., 2020; Deng *et al*., 2025; X. Wang *et al*., 2025). Such divergence in hormonal signalling is further evident in their transcriptional regulation and gene expression. For instance, quinoa showed reduced ABA accumulation, associated with the down-regulation of *PYL4* and *PP2C* (Fig. 7K, L; Table 2), while up-regulating ETH biosynthesis (*ACO*) and ETH response genes (*EIL5* and *ERF1*) (Fig. 7B–H; Table 2). This pattern is especially pronounced in the MZ and may contribute to the regulation of Na^+^ partitioning. Conversely, spinach exhibits a strong ABA response, marked by *PYL4* and *ASR1* up-regulation (Fig. 7K–N; Table 2). This observed ABA response aligns with a classic glycophytic strategy where ABA-mediated osmotic adjustment is prioritized (Sripinyowanich *et al*., 2013). This also aligns with our observed higher accumulation of proline and total phenols in spinach than in quinoa post-ROS treatment (Fig. 9E, F). While quinoa showed suppressed *PYL4* expression but increased ETH-related genes, especially at 24 h post-ROS stress in both root zones (Fig. 7), the previously reported ABA response in salt-grown quinoa compared with non-stressed quinoa plants (Rasouli *et al*., 2023) suggested divergent regulatory priorities between the two species under stress conditions. This also aligns with the previously established antagonistic interactions between ABA and ETH, affecting each other’s biosynthesis and signalling pathways (Müller, 2021). For instance, in Arabidopsis leaf development, loss-of-function mutations in *EIN2* and *EIN3*, as well as gain-of-function mutations in *ETR1* and *EIN4*, have been observed to confer salt sensitivity in mutants that accumulate higher endogenous ABA levels (Lei *et al*., 2011; Ueda *et al*., 2020). Conversely, inactivation of *CTR1* and *EBF1/EBF2* leads to enhanced salt tolerance and reduced ABA levels in Arabidopsis (Achard *et al*., 2006). Consistently, both species in our study adopt different strategies to confer oxidative stress tolerance/sensitivity.

Secondly, the temporal regulation of ETH-related genes under ROS underscores the adaptive advantage of quinoa: persistent *ACO* up-regulation may continuously drive ETH synthesis, while tissue-specific *EIL5* induction in the MZ (24 h post-ROS stress) is likely to fine-tune transporter activity to match the local ROS levels, indicating ROS-mediated ETH signalling as a conserved mechanism of K^+^ deficiency in halophytes (at least in quinoa) under high salinity (Zhang *et al*., 2020). Further, the robust MAPK signalling in quinoa, with higher *RBOHH* and *RBOHB* at 2 h and *MAPKKK20* at all time points (Fig. 6G, H), ensures coordinated ROS mitigation and transporter activation. In contrast, the fragmented MAPK activity of spinach (Table 2) could result in higher ROS production (Fig. 1C, D), higher ABA accumulation (Fig. 7J), and erratic ion transporter expression (e.g. suppressed *HAK5*, *SOS1*; Fig. 5), exacerbating K^+^ loss and Na^+^ toxicity.

Thirdly, such hormonal responses correlate with antioxidant dynamics: the transient APX/CAT activation in quinoa (peaking at 2 h; Fig. 9A–D) reflects efficient ROS mitigation, whereas the prolonged antioxidant up-regulation in spinach indicates chronic oxidative stress—a phenotype exacerbated by delayed antioxidant induction (24 h) (Fig. 9A–D). Consistent with this, a recent study showed that salt sensitivity in the glycophytic pepper *Capsicum chinense* was associated with reduced ETH signalling and MAPK signalling with higher oxidative burst compared with the halophytic pepper *Capsicum baccatum* (Farooq *et al*., 2024). Elevated ABA levels induce oxidative stress in plants (Majeed *et al*., 2023) and, in glycophytes such as Arabidopsis, maize, and rice, MAPK cascades reinforce ABA-induced antioxidant defences and proline accumulation to suppress ROS (Jammes *et al*., 2009; Shi *et al*., 2011; Sripinyowanich *et al*., 2013). Consequently, quinoa primarily up-regulated ETH signalling (Table 2; Fig. 7), with transcriptomes enriched in hormone-related pathways in its stress response (Ma *et al*., 2021). Concurrently, ETH pre-treatment enhanced the K^+^/Na^+^ ratio of quinoa by ∼27% in both root zones (Fig. 8A, B), underscoring the role of ETH in sustaining ion homeostasis, potentially via the ETH–MAPK network (Lang *et al*., 2020). Such differences highlight a fundamental trade-off: halophytes such as quinoa rely on ETH–MAPK networks for precise ion regulation, while glycophytes such as spinach depend on ABA-mediated damage control, sacrificing ion homeostasis for short-term survival. This mechanistic dichotomy explains the superior salinity tolerance of quinoa and underscores the importance of hormonal crosstalk in shaping stress adaptation strategies.

### Antioxidant zonation buffers reactive oxygen species for signalling, but not just for preventing oxidative damage

Salinity-induced oxidative stress disrupts cellular redox balance through excessive ROS production (Khan *et al*., 2020), yet the relationship between antioxidant capacity and stress tolerance remains nuanced. While constitutive ROS-scavenging systems were traditionally assumed to confer tolerance, studies show that antioxidant activity alone poorly correlates with salinity resilience (Jithesh *et al*., 2006; Maksimović *et al*., 2013; Tanveer and Shabala, 2018; Jia *et al*., 2023). Instead, spatiotemporal regulation of antioxidants appears critical, as excessive scavenging can disrupt ROS signalling essential for stress adaptation (Xu *et al*., 2025). This raises a key question: how do halophytic quinoa orchestrate tissue- and time-specific antioxidant responses to maintain redox balance and ionic homeostasis without compromising signalling?

In this context, our data revealed stark contrasts in ROS management between quinoa and spinach root zones. In quinoa, transient antioxidant activation (CAT) peaked at 2 h in the EZ—a region with inherently higher ROS (Bose *et al*., 2014; Jiang *et al*., 2016)—before declining by 24 h (Fig. 9A), mirroring its rapid ROS fluorescence dynamics (Fig. 1C, D). This response probably preserves ROS thresholds necessary for K^+^/Na^+^ regulation while preventing oxidative damage. Conversely, spinach exhibited sustained, systemic antioxidant up-regulation in both the EZ and MZ, reflecting chronic oxidative stress due to inefficient Na^+^ sequestration. Notably, the MZ of quinoa showed delayed antioxidant induction (Fig. 9B, D), suggesting that compartmentalized ROS signalling with rapid EZ responses may protect meristematic activity, while the MZ prioritizes vacuolar Na^+^ sequestration (reducing cytosolic toxicity and antioxidant demand)—a strategy analogous to redox priming in resurrection plants (Farrant, 2007). The failure of spinach to spatially restrict ROS production (Fig. 1D, E) probably disrupts tissue-specific signalling cascades.

Moreover, ROS levels (evidenced by cell viability) in the root meristem are several fold higher than in root MZs (Bose *et al*., 2014; Chakraborty *et al*., 2016; Jiang *et al*., 2016; Y. Liu *et al*., 2019), while in our study we observed that the timing of ROS production versus ROS scavenging was the most critical in both root zones. For instance, the abrupt antioxidant (CAT) surge at 2 h in quinoa aligns with halophytic models where H_2_O_2_ peaks early (e.g. *C. maritime* at 4 h; Ellouzi *et al*., 2011) before efficient scavenging restores homeostasis. The persistent antioxidant activity of spinach (Fig. 9), however, mirrors glycophytic *Arabidopsis thaliana*, where ROS accumulates unabated over 72 h (Ellouzi *et al*., 2011). This temporal divergence underscores a fundamental difference between both species: the phased response of quinoa allows ROS to act as a transient signal (e.g. for *SOS1* activation; L. Shabala *et al*., 2016), while the chronic oxidative load in spinach overwhelms signalling capacity. Critically, the tissue-specific antioxidant activity in quinoa, with rapid scavenging in the EZ versus delayed induction in the MZ—demonstrates how halophytes spatially segregate ROS functions, leveraging meristematic ROS sensitivity (Chakraborty *et al*., 2016; J. Liu *et al*., 2019) while protecting maturing tissues through vacuolar detoxification.

## Conclusion

Our study elucidates how tissue-specific ROS signalling orchestrates divergent K^+^/Na^+^ homeostasis in quinoa and spinach. The resilience of halophytic quinoa originates from its ability to (i) spatially segregate Na^+^ exclusion and sequestration; (ii) leverage hormone signalling for ion transport regulation; and (iii) dynamically balance ROS signalling and scavenging. The reliance of spinach on ABA signalling and global ROS suppression reflects a less coordinated response, leading to ionic imbalance. These insights advance the understanding of halophytic adaptation and highlight potential targets for improving salinity tolerance in crops, such as engineering tissue-specific transporter expression or modulating hormonal crosstalk. Though this study bridges the gap between ROS signalling and ion transport, questions remain. For instance, how do post-translational modifications (e.g. phosphorylation of *SOS1* by MAPKs) fine-tune transporter activity? Could the ETH-optimized responses of quinoa be engineered into glycophytes? Future work should explore single-cell transcriptomics to resolve tissue-specific regulatory networks and test genetic mutants to validate hub genes such as *EIL5* or *RBOHB*.

## Supplementary Material

erag021_Supplementary_Data

## Data Availability

The transcriptomic data generated in this study have been deposited in the National Center for Biotechnology Information (NCBI) Sequence Read Archive (SRA). For *C. quinoa*, the data are available under BioProject accession PRJNA1280883 (SRX29348900, SRX29348899). For *S. oleracea*, the data are available under BioProject accession PRJNA1280894 (SRX29352203, SRX29352202).

## Supplementary data

The following supplementary data are available at *JXB* online.

Fig. S1. An overview of global transcriptomics in roots under ROS stress.

Fig. S2. Expression of DEGs relating to phytohormone signalling under ROS stress.

Table S1. List of primers for qPCR.

Table S2. An overview of RNA-seq libraries.

Table S3. A list of significantly differentially expressed genes in quinoa under ROS.

Table S4. A list of significantly differentially expressed genes in spinach under ROS.

Table S5. List of enriched KEGG pathways in quinoa under ROS.

Table S6. List of enriched KEGG pathways in spinach under ROS.

Table S7. List of DEGs relating to ion homeostasis and phytohormone signalling in quinoa.

Table S8. List of DEGs relating to ion homeostasis and phytohormone signalling in spinach.

Table S9. List of DEGs relating to MAPK signalling in quinoa.

Table S10. List of DEGs relating to MAPK signalling in spinach.
