# Supplementary material for: Dissecting the ROS signalling component of salinity tolerance: tissue-specific K+/Na+ homeostasis in quinoa and spinach roots
Source: J Exp Bot. 2026 Jan 19;77(8):2468–89. doi: 10.1093/jxb/erag021 (PMC13080365; doi:10.1093/jxb/erag021)
Supplement: erag021_Supplementary_Data [file erag021_supplementary_data.zip › jexbot316967-file002.pdf]

Tissue-specific ROS signaling drives divergent K^+/Na^+ homeostasis in quinoa and spinach roots under salinity stress

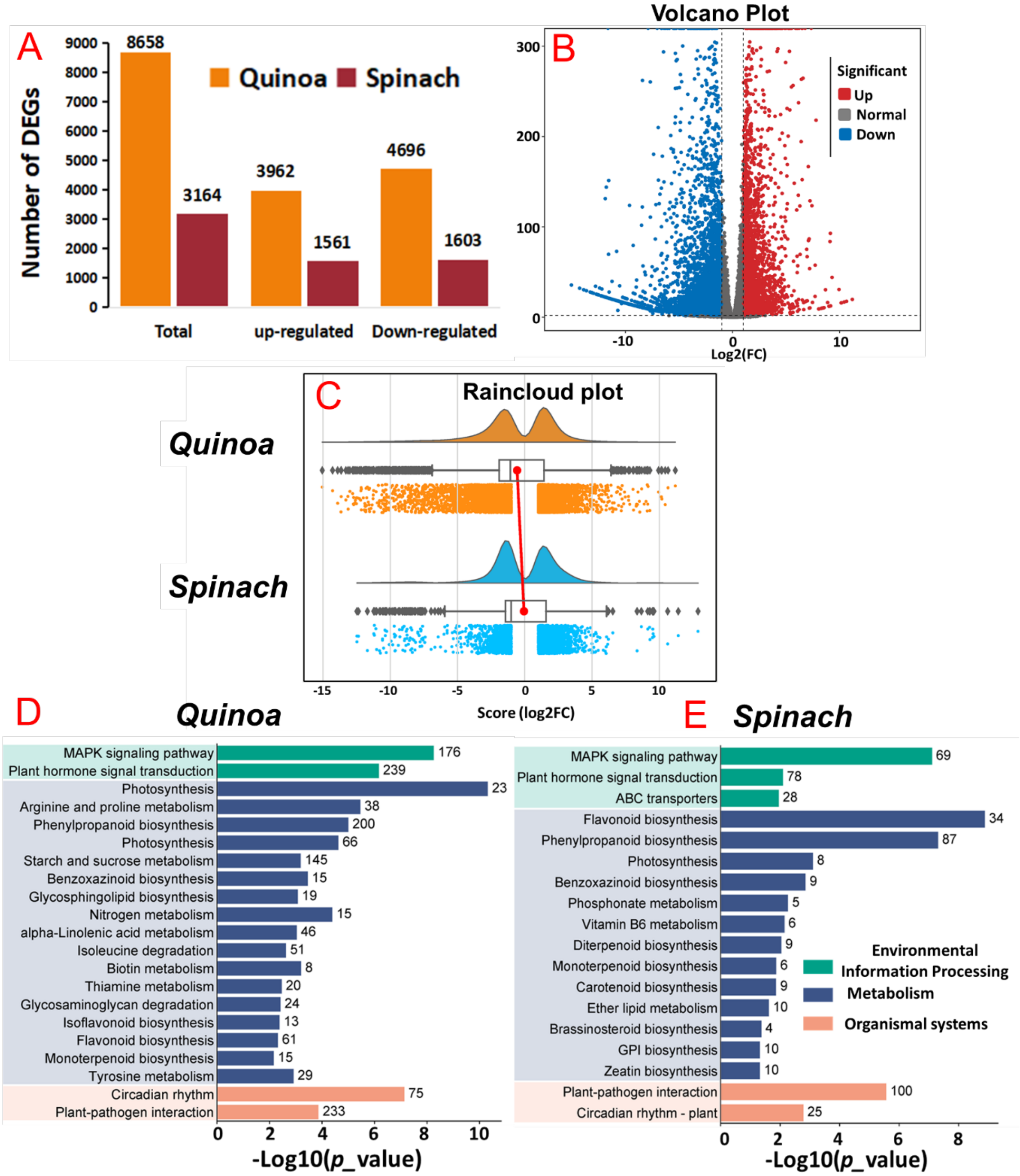

Supplementary Fig. S1: (A) Number of upregulated and downregulated DEGs from the roots of quinoa and spinach in response to ROS stress. (B) The volcano plot represents the upregulation and downregulation of DEGs. (C) The raincloud plot compares log2 fold change vs statistical significance (log10 p-value) for each gene represented. Panels (D) and (E) represent the top 20 KEGG pathways enriched in quinoa and spinach roots at 2h after ROS stress.

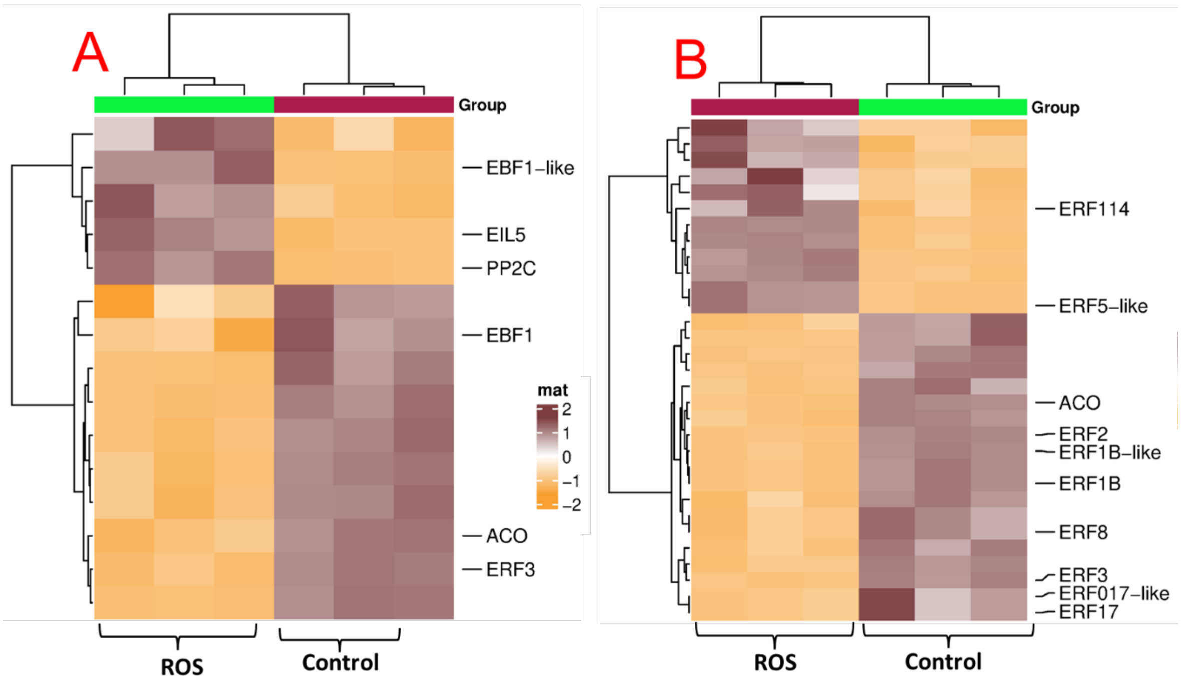

Supplementary Fig. S2: Heatmap analysis of the expression of DEGs relating to phytohormone signaling in (A) quinoa and (B) spinach at 2h post ROS stress.
